# Dynamics of the Microbiota in Response to Host Infection

**DOI:** 10.1371/journal.pone.0095534

**Published:** 2014-07-11

**Authors:** Clara Belzer, Georg K. Gerber, Guus Roeselers, Mary Delaney, Andrea DuBois, Qing Liu, Vera Belavusava, Vladimir Yeliseyev, Andres Houseman, Andrew Onderdonk, Colleen Cavanaugh, Lynn Bry

**Affiliations:** 1 Department of Pathology, Brigham & Women's Hospital, Boston, Massachusetts, United States of America; 2 Department of Pathology, Harvard Medical School, Boston, Massachusetts, United States of America; 3 Center for Clinical and Translational Metagenomics, Brigham & Women's Hospital, Harvard Digestive Diseases Center, Harvard Medical School, Boston, Massachusetts, United States of America; 4 Department of Organismic and Evolutionary Biology, Harvard University, Cambridge, Massachusetts, United States of America; 5 Department of Biostatistics, Harvard School of Public Health, Boston, Massachusetts, United States of America; Instutite of Agrochemistry and Food Technology, Spain

## Abstract

Longitudinal studies of the microbiota are important for discovering changes in microbial communities that affect the host. The complexity of these ecosystems requires rigorous integrated experimental and computational methods to identify temporal signatures that promote physiologic or pathophysiologic responses *in vivo*. Employing a murine model of infectious colitis with the pathogen *Citrobacter rodentium*, we generated a 2-month time-series of 16S rDNA gene profiles, and quantitatively cultured commensals, from multiple intestinal sites in infected and uninfected mice. We developed a computational framework to discover time-varying signatures for individual taxa, and to automatically group signatures to identify microbial sub-communities within the larger gut ecosystem that demonstrate common behaviors. Application of this model to the 16S rDNA dataset revealed dynamic alterations in the microbiota at multiple levels of resolution, from effects on systems-level metrics to changes across anatomic sites for individual taxa and species. These analyses revealed unique, time-dependent microbial signatures associated with host responses at different stages of colitis. Signatures included a *Mucispirillum* OTU associated with early disruption of the colonic surface mucus layer, prior to the onset of symptomatic colitis, and members of the Clostridiales and Lactobacillales that increased with successful resolution of inflammation, after clearance of the pathogen. Quantitative culture data validated findings for predominant species, further refining and strengthening model predictions. These findings provide new insights into the complex behaviors found within host ecosystems, and define several time-dependent microbial signatures that may be leveraged in studies of other infectious or inflammatory conditions.

## Introduction

Large-scale characterization of the host's microbiota has been enabled by recent innovations in sequencing technologies [1] and computational methods [2]–[4]. These developments have provided initial insights into the microbiota's association with normal physiology and disease [5]–[11]. Longitudinal studies are particularly valuable for unraveling causal interactions among the host and microbial inhabitants. However, studies of these ecosystems over time require new analytic approaches to fully explore their extraordinarily complex dynamics and identify signatures relevant to host outcomes [12], [13].

We used a mouse model of inflammatory colitis, caused by the attaching and effacing pathogen *Citrobacter rodentium* [14],[15], to investigate dynamic changes in microbial communities relative to a defined perturbation in the host. Prior studies have identified alterations in the gut flora at the height of acute infection [16]–[19], supporting our hypothesis that commensal populations change dynamically before and after onset of host symptoms, and may thus play important roles at different stages of disease. However, the kinetics of these changes are neither known, nor characterized. This experimental model thus provides a valuable system in which to discover the complex behaviors of the microbiota, across gut locations, and at different stages of host disease.

In immunocompetent mice *C. rodentium* infection follows four distinct stages [20]: 1) early colonization (≈1–6 days post-challenge), during which the pathogen establishes a small free-living reservoir in the cecum and ileum and initiates adherent infection in the distal colon [20], [21], 2) symptomatic infection (≈7–17 days), characterized by epithelial hyperplasia, influx of host immune cells, colitis, and development of early adaptive antibody responses [14], [22], 3) resolution (≈17–25 days), during which pathogen-specific IgG responses evolve and the pathogen is cleared from the host [23], and 4) convalescence (≈26–62 days), during which tissue damage is repaired.

We used high-throughput 16S rDNA gene sequencing to broadly characterize the microbiota over the course of infection. We also employed quantitative culture of the pathogen and predominant commensals to provide a complementary, non-nucleic acid based dataset. This approach allowed evaluation of the ecosystems under study at progressively finer levels of resolution, starting with systems-level properties, such as diversity and time to recovery, progressing to sets of taxonomic units, and lastly incorporating quantitative culture data to identify changes at the level of individual species.

Longitudinal analyses of complex microbial ecosystems present several computational challenges. First, the numbers of time-points and replicates collected from the host population(s) are frequently small due to sampling logistics and experimental costs. Second, biologic and analytic factors cause high amounts of noise. Third, limited sequencing depth, constrained by cost, and combined with relative rarity of certain organisms, can lead to low sequence counts for some taxa. Although a number of existing computational tools readily compare ecosystems' taxonomic compositions and diversities [2]–[4], or abundances of taxa between conditions [24], they have not been designed to analyze time-dependent changes in taxa relative to perturbations in the ecosystem. Tools for analyzing microarray time-series data [25]–[28] are also suboptimal for this latter application as their underlying algorithms do not model the characteristics of high-throughput sequencing data or microbiome data.

To address these challenges we extended a computational model that we recently developed, Microbial Counts Trajectories Infinite Mixture Model Engine (MC-TIMME) [29] to enable analysis of longitudinal changes in the microbiota during a host infection. MC-TIMME represents a new approach to analyzing microbiome time-series data, employing nonparametric Bayesian methods and continuous-time models of dynamics coupled with an error model tailored for high-throughput sequencing data. In prior work, we introduced the algorithm and applied it to a publicly available dataset measuring the microbiota of human subjects exposed to sequential antibiotic exposures [12]. In that work, we showed that MC-TIMME accurately inferred time-varying signatures for individual taxa while simultaneously compressing similar signatures into groups. Our method further identified a number of new features in the dataset that had not been found using standard analysis techniques. These new findings included characterization of relaxation time distribution, or the kinetics of ecosystems' return to baseline or new levels after introduced perturbations, and discovery of consensus signature groups (CSGs), which represent sets of reference OTUs within or among subjects that share common behaviors over the time-series. In the present work, we extend MC-TIMME with a new, flexible model of dynamics to capture behavior of the microbiota during an ongoing host infection and introduce methods for incorporating complementary data sources into analyses, including quantitative culture data.

Application of the extended version of MC-TIMME to our datasets of 16S rDNA gene signatures and quantitatively cultured isolates from mice infected with *C. rodentium* enabled study of host microbial ecosystems during an infection at progressively finer levels of resolution. First, we analyzed time-dependent changes in systems-level properties of the intestinal ecosystems and found substantial differences across anatomic sites. Second, we used Consensus Signature Group (CSG) analyses to characterize the range of time-varying signatures observed in the microbiota subsequent to host infection with the pathogen. Third, we generated time-maps to temporally order CSG dynamics and to visualize coordinate and cascading changes contributed by individual taxa across intestinal sites. Lastly, we incorporated quantitative culture data for the pathogen and predominant commensals to validate and refine model predictions of the dynamics observed, providing the final component of our ecosystem-to-species level discovery of temporal dynamics in the microbiota during a host infection.

## Results

### High-throughput longitudinal profiling of the microbiota during host infection

**Figure 1:**
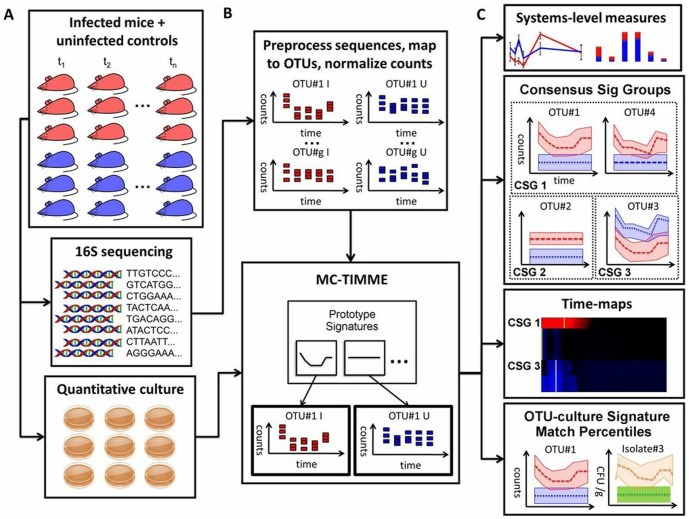
Experimental and computational framework. **(A)** Experimental model, with cohorts of infected mice (red) or uninfected age-matched controls (blue), from which ileum, cecum and distal colon samples were collected. Samples were subjected to high-throughput 16S rDNA sequencing as well as quantitative culture for the pathogen and predominant commensals. **(B)** Computational pipeline begins with preprocessing and clustering of 16S rDNA sequences into Operational Taxonomic Units (OTUs). Normalized OTU or culture counts serve as input to MC-TIMME, which simultaneously infers the number, shapes, and assignments of taxa to prototype signatures. **(C)** MC-TIMME outputs summarize dynamic changes in microbial communities across intestinal sites at multiple levels of detail. Systems measures detect large-scale changes in microbial community structure and dynamics. Consensus Signature Groups (CSGs) compress OTUs into sets with members exhibiting similar behaviors over time. Time-maps compactly visualize microbiota dynamics in tissues, organizing CSGs by their times of maximal change to reveal cascades of coordinate alterations. Signature Match Percentiles (SMPs) identify taxa for which sequence and culture-derived signatures have strong correspondences.

To explore the dynamic effects of *C. rodentium* infection on intestinal ecosystems, samples from ileum, cecum and distal colon were collected from infected mice and uninfected controls, at days 3, 7, 10, 14, 21, 28 and 62 post-challenge with the pathogen. Each time point consisted of biological replicates for the infected or uninfected groups. Samples were subjected to massively parallel 16S rDNA gene sequencing and quantitative culture using media selective for the pathogen and predominant commensals (Fig. 1A, Tables 1, 2, Table S1, Datasets S1, S2).

**Table 1:** Summary of sequenced samples and Good's coverage estimates.

	T	N (ctrl)	N (inf)	Reads (ctrl) K	Reads (inf) K	Coverage (ctrl)	Coverage (inf)
**Ileum**	3	3	3	2.9–3.6	0.8–3.6	98–100%	98–99%
	7	3	3	3.1–4.0	3.7–4.3	95–99%	98–99%
	10	3	3	2.2–3.3	1.9–3.9	98–99%	97–99%
	14	3	2	3.4–4.5	4.7–5.6	97–99%	99–100%
	28	3	3	2.7–2.9	0.8–3.0	96–98%	92–96%
	62	3	3	4.2–5.8	3.8–4.2	96–98%	97–99%
**Cecum**	3	3	4	1.0–1.3	1.1–2.4	84–98%	88–93%
	7	3	3	0.7–0.8	0.7–1.2	70–76%	83–88%
	10	3	3	2.0–2.6	2.3–2.6	88–92%	92–95%
	14	3	3	1.1–1.3	1.0–1.3	77–79%	74–78%
	28	3	3	2.0–4.4	1.7–2.3	87–90%	85–89%
	62	3	3	1.8–2.1	1.6–1.8	81–85%	79–82%
**Colon**	3	3	3	3.5–4.6	3.4–3.7	93–100%	97–100%
	7	2	2	2.8–3.9	2.6–2.9	95–99%	99–100%
	10	3	3	0.8–2.5	2.4–3.6	90–96%	98–99%
	14	1	2	2.8	2.8–2.9	96%	99–100%
	28	4	3	1.9–3.2	2.3–2.5	94–98%	87–91%
	62	3	2	1.0–6.9	1.5–6.1	94–98%	96–99%

Numbers of reads are listed in thousands, and are after all preprocessing quality control and filtering steps. The average number of reads across all samples was ≈2,700. Good's coverage is a nonparametric estimate of the proportion of classes (i.e., OTUs) that are observed in a sample out of the total number of classes inferred to be present in the population. Labels in the top table row: T  =  time-point, ctrl  =  control, uninfected mice; inf  =  infected mice.

**Table 2:** Species identified by quantitative culture.

Species	Selective Media	Growth Conditions	Identification	Threshold of detection	16S rDNA gene ID
***Citrobacter rodentium***	MAC	Aeroboic^1^, 24 hr	API-20E panel and full 16S sequence.	100 CFU/g	*Citrobacter rodentium*
***Enterobacter hormachei***	MAC	Aeroboic^1^, 24 hr	API-20E panel and full 16S sequence.	100 CFU/g	*Enterobacter hormachei*
***Lactobacillus johnsonii***	CNA	Anaerobic^2^, 48–72 hr.	Biochemical typing and 16S sequence	500 CFU/g	*Lactobacillus johnsonii*
***Lactobacillus murinus***	BKV and CNA	Anaerobic^2^, 48–72 hr.	Biochemical typing and 16S sequence	100 CFU/g	*Lactobacillus murinus*
***Lactobacillus reuteri***	CNA	Anaerobic^2^, 48–72 hr.	Biochemical typing and 16S sequence	500 CFU/g	*Lactobacillus reuteri*
***Proteus vulgaris***	MAC+TET	Aeroboic^1^, 24 hr	API-20E panel and full 16S sequence.	100 CFU/g	*Proteus vulgaris*

Agar media used: MAC (MacConkey agar) for selection of enteric and bile-resistant non-fermenter species; MAC+TET (MacConkey agar with 10 µg/mL of Tetracycline) to select for *Proteus vulgaris*. CAN (Colistin Naladixic Acid agar with 5% sheep's blood on a Columbia agar base) for suppression of enteric species; BKV (Brucella-Kanamycin-Vancomycin agar) for selection of aminoglycoside+vancomycin-resistant Lactobacilli;

1 Aerobic incubation conditions were in 5% CO_2_ humidified atmosphere at 37°C; plates read at 24 and 48 hours of incubation.

2 Anaerobic conditions were in a Coy anaerobic chamber at 37°C. Plates were read in the chamber at 72 hours of incubation.

We calculated Good's coverage estimator [30] (Table 1) for sequenced samples to determine if sequencing coverage was equivalent between infected and uninfected mice, particularly in distal colon where large pathogen burdens (maximum of ≈99% of sequencing reads in the colon, and ≈4% of sequencing reads in cecum and ileum) could impact ability to detect shifts in the underlying microbiota. Average coverage was lowest in the more microbiologically diverse cecal samples at ≈85%, and highest, at ≈95–98% for colon and ileum. However, infected and uninfected mice demonstrated comparable coverage at each location, indicating that introduction of the pathogen did not prevent detection of underlying commensal populations in infected mice.

### Robust inference of time-varying signatures of taxa

We applied an extended version of MC-TIMME to our dataset to infer time-varying signatures of taxa. Replicated time-series of counts, either of Operational Taxonomic Units (OTUs) derived from sequencing data, or of individual species measured by quantitative culture, served as input to MC-TIMME (Fig. 1B). MC-TIMME uses nonparametric Bayesian methods to simultaneously estimate the number of signatures, the shapes of signatures, and assignments of taxa to signatures (Fig. 1C). Taxa may follow different signatures in distinct anatomic sites and in the infected and uninfected states, which our algorithm automatically detects [29]. From the 16S rDNA data, 210 OTUs had sufficient counts for analysis. Of these, MC-TIMME identified 45 OTUs with detectable changes in response to infection in at least one intestinal site. For the culture data, out of 7 predominant commensal species determined to have sufficient counts for analysis across biological replicates, all demonstrated detectable changes in response to infection in at least one site. The inferred trajectories formed the basis for subsequent analyses at ecosystems-to-species levels of detail (Fig. 1C).

### Ecosystems-level measures: dynamics of microbiota diversity and ecosystem recovery times

**Figure 2:**
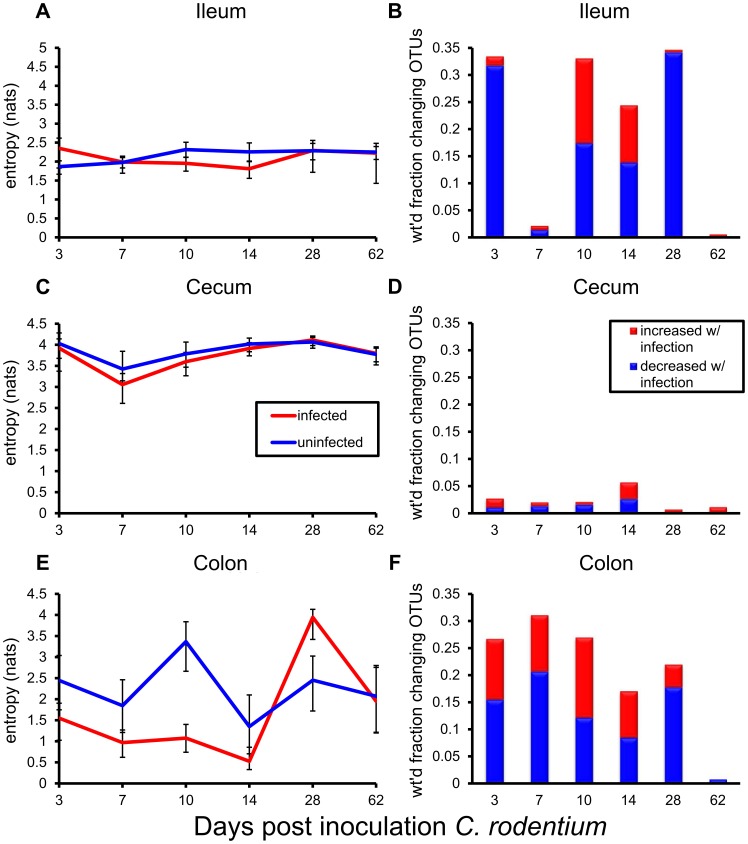
Systems-level measures of microbiota diversity dynamics and tissue recovery times in response to infection. (**A, C, E**) Shannon entropy calculated from inferred Operational Taxonomic Unit (OTU) signatures measures dynamic changes in the diversity of the microbial ecosystems in each tissue. Red line  =  entropy of infected tissue ecosystem; blue line  =  entropy for controls. Vertical bars denote 95% credible intervals. (**B, D, F**) The Microbiota Recovery Time (MRT) in each tissue measures the latest time-point post-challenge with the pathogen for which microbial communities from infected mice and controls exhibit >95% similarity overall. Red bars  =  weighted measure of detected changes in taxa increasing with infection; blue bars  =  corresponding measure for decreasing taxa. The weighted measure takes into account relative taxa abundances in both infected and uninfected cohorts. The recovery time was 62 days for ileum, 14 days for cecum, and 62 days for colon.

We used inferred signatures to estimate an ecosystem-level measure of microbiologic diversity, Shannon entropy [31], and to detect changes in this measure over the infection. In both groups of mice, the Shannon entropy measure indicated that cecum had the highest levels of diversity over the time series (Fig. 2C), followed by colon (Fig. 2E), and ileum (Fig. 2A). Diversity decreased with infection across all gut locations, with nadirs occurring over days 7–14, the period of symptomatic infection. Of note, the most profound decreases occurred in distal colon at the primary site of infection. However, by 2 months post-challenge, overall diversity in the infected mice returned to that of uninfected mice at all gut locations.

We next evaluated the time it took for each ecosystem to return to baseline after introduction of the pathogen. To quantify this duration, we developed a new measure, the Microbiota Recovery Time (MRT). The MRT is defined as the latest time-point post-challenge with *C. rodentium* for which microbial communities from infected mice and controls exhibit no detectable differences (>95% similarity). For the MRT, the calculated similarity between microbial communities takes into account the relative taxa frequencies derived from their inferred signatures (Protocol S1). The estimated MRT was 62 days for ileum, 14 days for cecum, and 62 days for colon. This analysis highlighted very different patterns of change among gut locations over the course of the infection as well as the time required for recovery to a stable state. Ileum (Fig. 2B) showed a biphasic pattern with prominent increases of taxa abundances during early infection followed by decreases during acute infection. The microbiologically diverse cecum showed little detectable change throughout infection (Fig. 2D), while colon showed uniform decreases in early infection that resolved after the recovery phase (Fig. 2F).

### Consensus Signature Groups: defining patterns of commensal responses to infection

**Figure 3:**
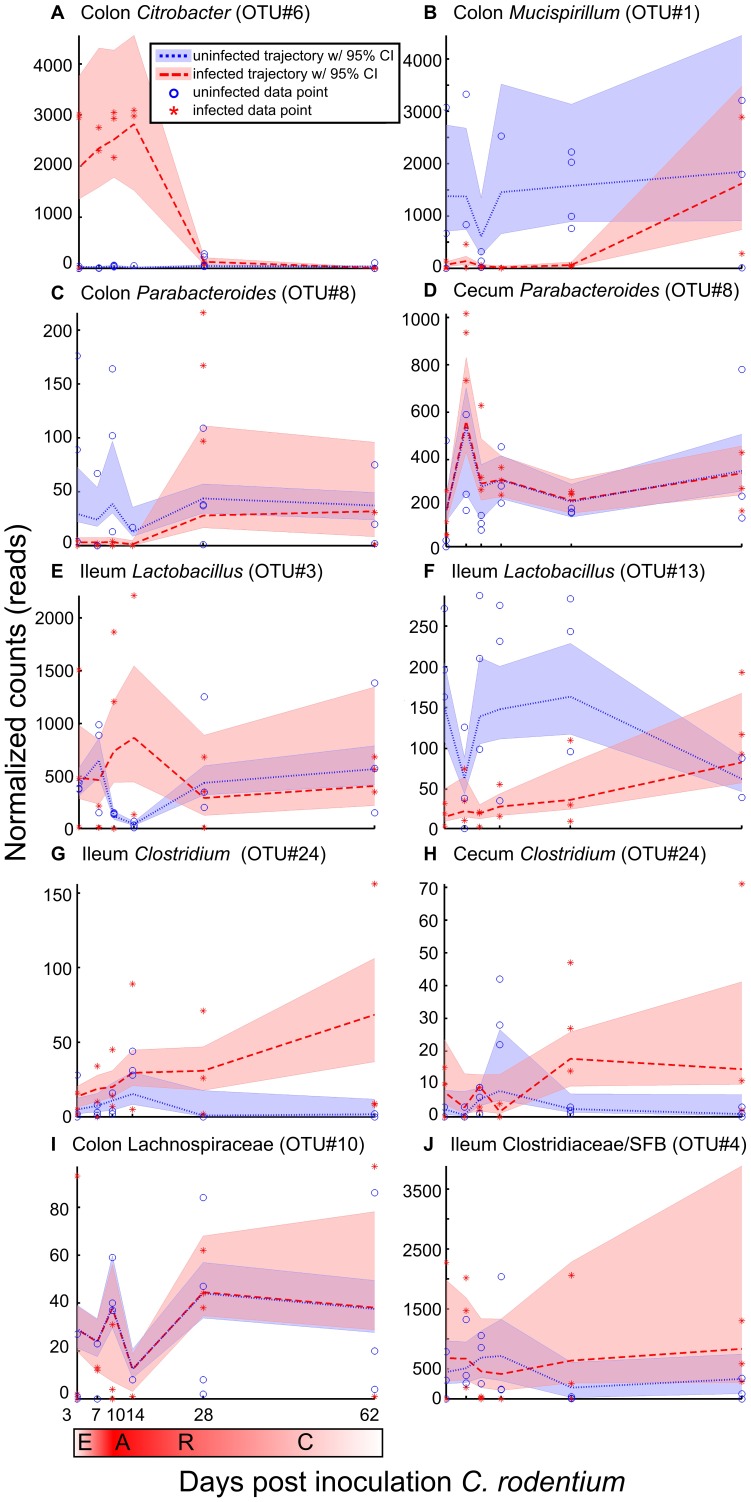
Consensus Signature Groups systematically characterize patterns of time-dependent microbiota changes in response to infection. Consensus Signature Groups (CSGs) represent sets of taxa that share similar dynamics within a tissue, providing a means to identify common behaviors among taxa regardless of their phylogenetic relationships. Representative signatures of individual Operational Taxonomic Units (OTUs) from CSGs are shown. Horizontal axis indicates days post-inoculation with the pathogen; vertical axis shows normalized sequencing counts for the OTU. Dashed or dotted lines indicate median signature shapes for OTUs. Shaded regions indicate 95% credible intervals for signatures; regions of overlap indicate time-periods during which changes were not detected. Phases of infection are E  =  early, A  =  acute, R  =  recovery, C  =  convalescence. (**A**) The pathogen, *Citrobacter rodentium* (OTU#6) in colon. (**B**) *Mucispirillum* (OTU#1) in colon, rapidly decreases and does not return to baseline until the convalescent phase. (**C**) *Parabacteroides* (OTU#8) in colon, decreases during early infection, but returns to baseline by the recovery phase. (**D**) *Parabacteroides* (OTU#8) in cecum had no detectable change between cohorts. (**E–F**) Two *Lactobacilli* in ileum, showing different dynamics: OTU#3 increases during acute infection, while OTU#13 decreases. (**G–H**) *Clostridium* (OTU#24) in ileum and cecum, has a delayed increase that persists into the convalescent phase. (**I–J**) Representative OTUs in colon and ileum showing no detectable changes between cohorts.

**Figure 4:**
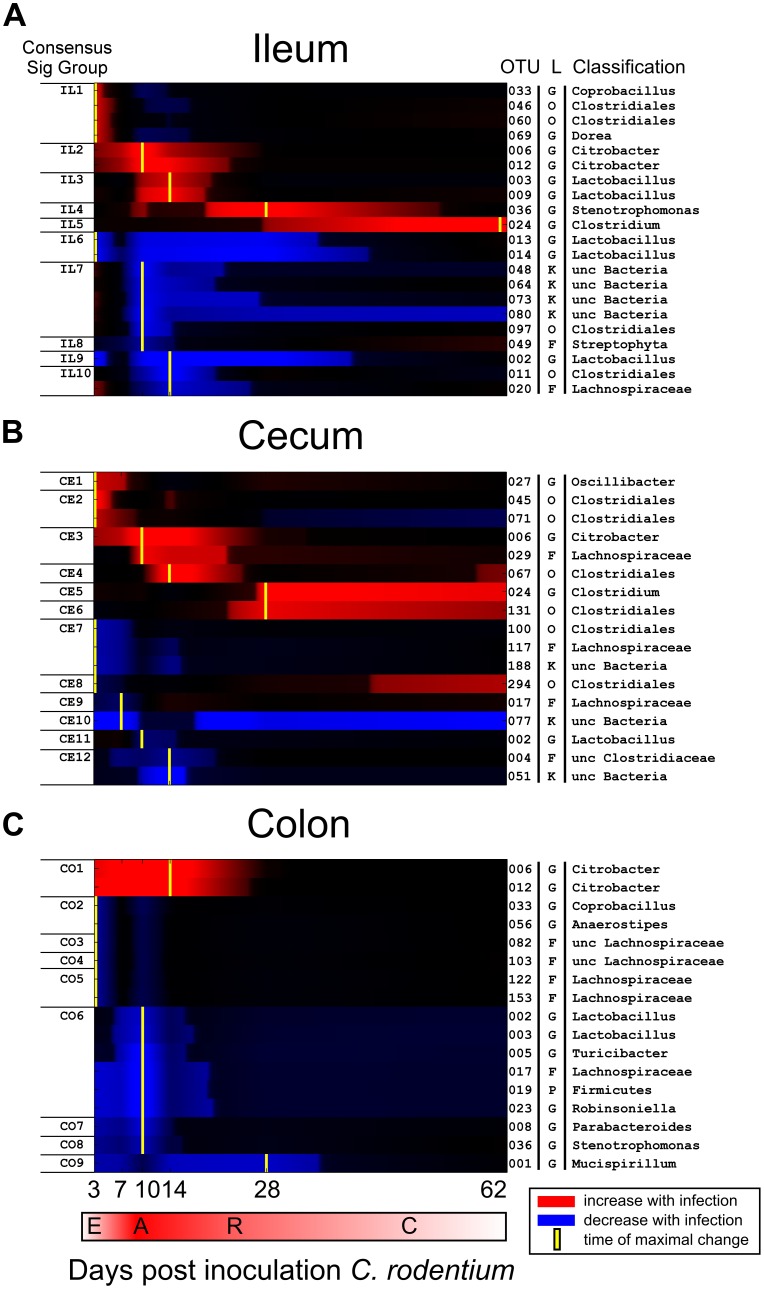
Time-maps reveal tissue-specific cascading and coordinate changes among commensal taxa in response to infection. MC-TIMME detected 45 Operational Taxonomic Units (OTUs) that change over time between infected and uninfected mice in at least one intestinal site, and assigned these OTUs to Consensus Signature Groups. Time-maps use CSG information to visualize interrelations among the dynamic responses of OTUs within intestinal ecosystems. X-axis indicates days post-challenge with *C. rodentium*. Rows in each panel depict OTUs with detectable changes in infected mice relative to uninfected controls. Red indicates an increase in the signature for infected mice relative to baseline, and blue indicates a corresponding decrease. Intensity of colors corresponds to magnitude of change of signatures; regions in which changes were not detected are attenuated in color. Yellow marks indicate the time of maximal increase or decrease relative to the uninfected baseline controls for the CSG. Note that magnitudes of changes are not directly comparable across OTUs in this visualization. Phases of infection are E  =  early, A  =  acute, R  =  recovery, C  =  convalescence. Vertical axis on the right-hand side indicates the OTU#, level of taxonomic assignment by the Ribosomal Database Project (RDP) classifier (L  =  level; K  =  kingdom; P  =  phylum; O  =  order; F  =  family; G  =  genus), and RDP taxonomic assignment.

We next used Consensus Signature Groups (CSGs) to categorize the types of dynamic changes that occurred in the gut microbiota after introduction of the pathogen (Figs 3,4). A Consensus Signature Group represents a set of taxa that share similar dynamics within a tissue, providing a means to identify common behaviors among taxa regardless of their phylogenetic relationships. All 45 OTUs identified by MC-TIMME as having detectable changes in response to infection in at least one intestinal site were assigned to CSGs.

Figure 3 illustrates representative signatures discovered by our method for individual OTUs. These OTUs belong to CSGs that exemplify predominant patterns of change in the ecosystems studied. As expected, the pathogen *Citrobacter rodentium* in colon (OTU#6; Fig. 3A), shows a rapid increase with maximal change during acute infection, and returns to baseline by the resolution phase. In contrast, *Mucispirillum* in colon (OTU#1; Fig. 3B), demonstrates a rapid decrease during early infection. This decreased exhibits a prolonged delay to recovery, beyond the period of pathogen clearance between days 21–28. Of note, *Parabacteroides* in colon (OTU#8; Fig. 3C) also decreased during early infection, but returns to baseline by the recovery phase when the pathogen has been cleared. *Lactobacillus* (OTU#3; Fig. 3E) in ileum shows an increase that occurs during acute infection, and then quickly returns to baseline by the recovery phase, whereas a second *Lactobacillus* (OTU#13; Fig. 3F) shows an immediate and prolonged decrease in the ileum. In contrast, *Clostridium* (OTU#24) in ileum (Fig. 3G) and cecum (Fig. 3H), shows a very delayed increase that persists into the convalescent phase.

Of note, many taxa demonstrated no detectable differences between infected and uninfected mice, highlighting the fact that profound changes in host microenvironments may preferentially affect select taxa. For instance, *Parabacteroides* (OTU#8), which showed changes in the colon (Fig. 3C), had no detectable changes at its predominant site of residence in the cecum (Fig. 3D). Other examples of taxa that showed no differences between infected and uninfected mice include a member of family Lachnospiraceae in colon (OTU#10; Fig. 3I) and a member of family Clostridiaceae in ileum (OTU#4; Fig. 3J), which further classified as segmented filamentous bacteria (SFB) by the RDP Sequence Match tool [32].

The analysis of Consensus Signature Groups suggested that a substantial number of taxa in each tissue responded to infection in a coordinate manner over the 2-month period. To quantify this effect, we developed a measure, Consensus Signature Group Coordination (CSGC), which is one minus the ratio of the number of CSGs identified in a tissue to the number of taxa present in that location across the time-series. CSGC thus measures the degree of coordination of the changes among taxa within the microbial tissue ecosystem. Higher CSGC values indicate more coordinated behavior, while lower values indicate more varied responses. The CSGC values for our dataset were 83% in ileum, 92% in cecum, and 90% in colon. These values indicate highly coordinated changes in the microbiota within all intestinal ecosystems during infection, but with ileum showing notably more varied changes than cecum or colon.

### Time-maps: identifying tissue-specific, cascading changes among commensal taxa

We created time-maps to visualize interrelations among the dynamic responses observed for taxa within intestinal ecosystems (Figs 4A, 4B, 4C). Time-maps revealed cascading and coordinate changes across all gut locations studied, identifying distinct patterns within each tissue.

In ileum (Fig. 4A), groups of OTUs coordinately increased or decreased in response to infection, with the majority showing two waves of peak responses. The first peak occurred early during infection, over days 3–7 post-challenge (CSG IL1 and IL6), while the second wave occurred during acute symptomatic infection over days 10–14 post-challenge (CSGs IL2, IL3, IL7–IL10). Overall, dominant effects in infected mice were seen in the orders Clostridiales and Lactobacillales within phylum Firmicutes, though many OTU classifying at the same taxonomic level exhibited quite different dynamics. In infected mice, OTU associated with the genus *Lactobacillus* both increased (OUT#3, #10) and decreased (OTU#13, #14), while members of the *Clostridiales* increased (OTU#33, *Coprobacillus*; OTU# 46, #60, Clostridiales; and OTU#69, *Dorea*) and also decreased (OTU#11, Clostridiales).

In cecum (Fig. 4B), although the *Citrobacter* signature comprised <≈5% of total reads per sample, even at the height of infection, numerous OTUs demonstrated altered trajectories in infected mice. Members of family Lachnospiraceae both increased (OTU#29) and decreased (OTUs #117 and #017) in infected mice as compared to uninfected controls. Within the *Clostridiales* OTUs #045 and #071 (CE2) increased in infected mice only over the first 7 days of infection. In contrast, OTU#67 increased over early and acute stages of infection (CSGs CE4), while OTUs corresponding to genus *Clostridium* and that typed taxonomically to other members of the Clostridiales (OTU#24, 131 and 294), increased after the pathogen's clearance (CSGs CE5, CE6, CE8). Among the Lachnospiraceae, OTU #29 (CE3) increased over acute infection, while OTU#117 (CE7) declined during early stages of colonization, as did OTU #017 (CE9). In contrast, these taxa showed nominal variation over the course of infection in uninfected mice.

In colon (Fig. 4C), other than the pathogen, OTUs with detectable changes showed profound decreases in infected mice, with many CSGs exhibiting a time of maximal decrease by day 3 (CSGs CO2–CO5), prior to the onset of symptomatic infection. Affected taxa included *Anaerostipes* (OTU#56) and members of the Lachnospiraceae (OTUs#82, 103, 122 and 153). A second wave of affected CSGs showed maximal decrease by day 10 during symptomatic infection (CSGs CO6–CO8). Both waves of affected CSGs largely recovered by the time of pathogen clearance at day 28. Genus *Mucispirillum* in colon (OTU#1; CSG CO9) was an exception, exhibiting a sustained decrease into the convalescent phase, beyond *C. rodentium'*s clearance by day 28. Of the tissues studied, the colon demonstrated the most phylogenetically diverse set of OTUs that changed in response to infection, including OTUs classifying at the genus level as *Mucispirillum* (phylum Deferribacteres), *Robinsoniella*, *Lactobacillus*, *Turicibacter* (phylum Firmicutes) and *Parabacteroides* (phylum Bacteroidetes).

### Validating predictions to the species level with complementary data sources

We used quantitative culture of predominant species to validate dynamics of corresponding taxa identified with high-throughput sequencing. Sequence and culture-based datasets provide complementary information. With sequence-based counts the prevalence of a given OTU must be interpreted relative to the total counts for all OTUs in the ecosystem. In contrast, quantitative culture normalizes counts for a species relative to the input mass of tissue. By measuring viable organisms, culture-based analyses are not confounded by the presence of nucleic acid signatures from large numbers of dying organisms, which could occur with pathogen clearance. Thus, although culture based methods cannot be used to broadly characterize a complex ecosystem, selective use provides an alternate and sensitive method for measuring changes in defined species. The presence of common signals in both data sources for a given taxon, and corresponding species, provides stronger evidence that the signatures reflect the underlying dynamics *in vivo*.

**Figure 5:**
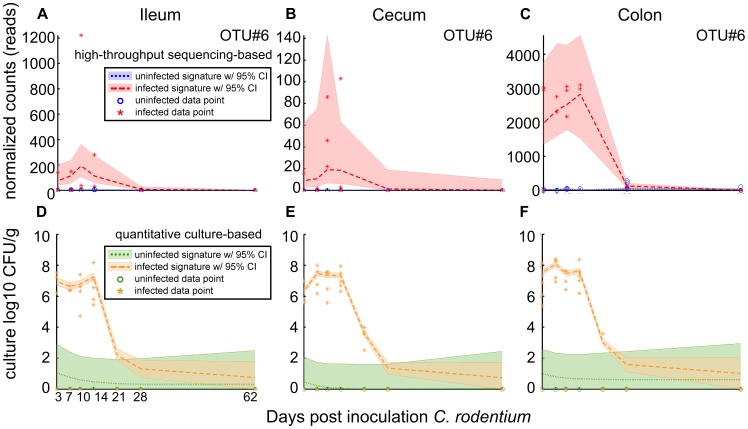
Sequence and culture-based signatures for *Citrobacter rodentium* in all tissues. Horizontal axis indicates days post-inoculation with *C. rodentium*. Dashed lines indicate the inferred median signature shape for each trajectory. Shaded regions indicate the 95% credible interval. **(A, B, C)** Signatures derived from sequencing data for the predominant *C. rodentium* Operational Taxonomic Unit (OTU) in ileum, cecum, and colon. Vertical axis indicates the number of normalized sequencing counts. **(D, E, F)** Signatures derived from culture-based data for *C. rodentium* in ileum, cecum and colon. Vertical axis indicates log_10_ Colony Forming Units (CFUs) per gram of input tissue. Of note, although *C. rodentium* was not cultured from uninfected mice, the estimated upper bound of the 95% credible interval for the culture counts in uninfected and infected mice trends to 1X10^2^ CFU/g, which was the threshold of detection when using MacConkey agar for selective culture (Table 3).

**Figure 6:**
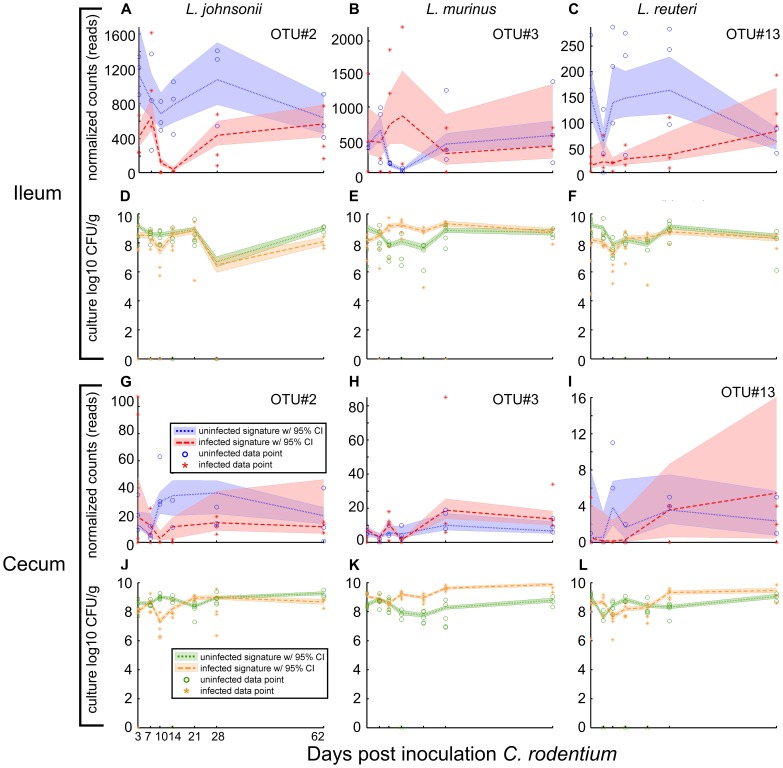
Sequence and culture-based signatures for three *Lactobacillus* species in ileum and cecum. Horizontal axis indicates days post-inoculation with *Citrobacter rodentium*. For sequence-based signatures, vertical axis indicates the number of normalized sequencing counts for the Operational Taxonomic Unit (OTU). For culture-based signatures, vertical axis indicates log_10_ Colony Forming Units (CFUs) per gram of input tissue. Dashed lines indicate the inferred median signature shape for each trajectory. Shaded regions indicate the 95% credible interval. **(A, G)** Sequence and **(D, J)** culture-based signatures for *Lactobaillus johnsonii*. **(B, H)** Sequence and **(E, K)** culture-based signatures for *Lactobacillus murinus*. **(C, I)** Sequence and **(F, L)** culture-based signatures for *Lactobacillus reuteri*.

In addition to sequence and culture-based dynamics detected for *C. rodentium* (OTU#6; Fig 5), MC-TIMME detected altered ileal or cecal dynamics in response to infection for five OTUs, (#2, 3, 9, 13 and 14), which the RDP Classifier [33] classified to the genus *Lactobacillus*. These OTUs proved resolvable to the species level with the RDP Sequence Match tool (34). OTU#2 classified as *L. johnsonii*, OTUs#3 and 9 as *L. murinus*, and OTUs#13 and 14 as *L. reuteri*. Interestingly, quantitative culture of predominant organisms identified these same species, and we applied MC-TIMME to generate signatures based on the culture counts (Fig 6).

To evaluate the similarity of signatures inferred from sequence and culture-based datasets, we calculated the rank of the match between the culture-based signature and the corresponding OTU signature, relative to all other OTU signatures in the system (the Signature Match Percentile, SMP). This measure provides a principled way to leverage alternate methods for measuring individual members within a complex ecosystem, such as quantitative culture or use of a different sequencing methodology, to validate findings identified by the originally used method. SMP values >50% indicate specific matches between the culture and sequence-based signatures. In contrast, SMP values <50% indicate relatively non-specific matches between the culture and sequence-based signatures, raising the possibility that the observed correspondence may be due to chance alone, and thus may be less reflective of true *in vivo* behaviors.

The three *Lactobacillus* species exhibited specific matches between culture and sequence-derived signatures for each organism in ileum and cecum (Table 3). In both sources of data, *L. johnsonii* exhibited a *decrease* during acute infection (79th-centile SMP in ileum and 80th-centile SMP in cecum; Fig. 6A), *L. murinus* exhibited an *increase* during acute infection (94th-centile SMP in ileum and cecum; Fig. 6B), and *L. reuteri* exhibited a *decrease* in acute infection (91th-centile SMP in ileum and 80th-centile SMP in cecum; Fig. 6C). The high SMP values indicate that the identified signatures are in close agreement between the two data sources, suggesting that each *Lactobacillus* species may play different functional roles within the ileal and cecal ecosystems.

**Table 3:** Correlation of Sequence and Culture-based Signatures.

Tissue	Organism	Uncentered correlation of culture vs. sequence signatures	Percentile of correlation
**Ileum**	*C. rodentium*	0.82	98%
	*L. johnsonii*	0.59	79%
	*L. murinus*	0.66	94%
	*L. reuteri*	0.69	91%
**Cecum**	*C. rodentium*	0.82	98%
	*L. johnsonii*	0.63	80%
	*L. murinus*	0.76	94%
	*L. reuteri*	0.50	80%
**Colon**	*C. rodentium*	0.89	>99%

### MC-TIMME detects differing signatures among low abundance commensal species phylogenetically related to *C. rodentium*

**Figure 7:**
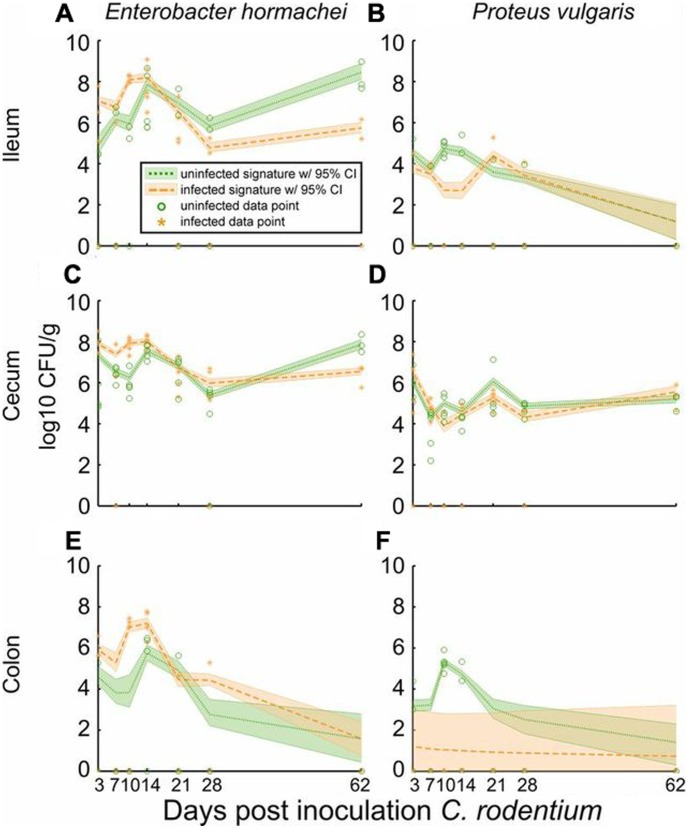
Culture-based signatures for low abundance commensal species phylogenetically related to *Citrobacter rodentium* in ileum, cecum, and colon. Horizontal axis indicates days post-inoculation with *C. rodentium*. Vertical axis indicates log_10_ Colony Forming Units (CFUs) per gram of input tissue. Dashed lines indicate the inferred median signature shape for each trajectory. Shaded regions indicate the 95% credible interval. **(A, C, E)** Signatures for *Enterobacter hormachei*. **(B, D, F)** Signatures for *Proteus vulgaris*.

*Enterobacter hormachei* and *Proteus vulgaris*, both members of family Enterobacteriaceae, normally attain their highest biomass in the mouse cecum where they comprise a minor (10^3^–10^5^ CFU/g), but consistently present, population of the ecosystem involved in rodent hindgut metabolism [34]. With 2000–3000 sequencing reads/sample these organism are effectively undetectable. Thus, we used culture-based methods and input these counts into MC-TIMME to gain an understanding of the dynamics of these organisms during the *C. rodentium* infection. *E. hormachei* showed a detectable increase then decrease that correlated with the signature of *C. rodentium* (Fig 7A, C, E). In contrast, *P. vulgaris* demonstrated a detectable decrease in ileum and distal colon (Fig 7B, 7F), though negligible effects in cecum (Fig 7D).

## Discussion

Our study demonstrates the power of using an integrated experimental and computational approach to analyze complex microbial ecosystems over time. Utilization of a defined experimental system enabled collection of high-quality samples from multiple intestinal sites, and reduced confounding factors though use of age-matched controls and biological replicates. Our computational model was designed to effectively leverage sparse and noisy time-series of counts produced from high-throughput sequencing and quantitative culture data. These approaches enabled robust detection of temporal changes in the behavior of the microbiota at multiple levels of resolution. Furthermore, this approach characterized reproducible changes in predominant organisms to the level of culturable isolates, illustrating the utility of combining molecular, microbiologic and computational methods for study of dynamically changing microbial ecosystems.

Many of the time-dependent microbial signatures discovered by our analyses are associated with key events in the host response to *C. rodentium* infection. For example, Consensus Signature Group (CSG) CO9, represented by the genus *Mucispirillum* (OTU#1) in colon, showed a prolonged decrease in infected mice. *In vivo*, *Mucispirillum* inhabits the mucus layer over colonocytes. *C. rodentium*'s adherence to colonic epithelium actively destroys this microenvironment [35]–[37]. Full regeneration of the mucus layer occurs some time after the pathogen's clearance, providing a possible explanation for the observed delay in *Mucispirillum*'s recolonization of distal colon. This CSG could thus provide a marker for health of the surface mucus layer in distal colon, with potential application to other models of inflammatory colitis. As another example, MC-TIMME identified multiple CSGs exhibiting maximal decreases in infected mice over the second and third weeks post-challenge. This timeframe covers the period of maximal pathogen burden in the distal colon, the height of host symptoms, and the early development of pathogen-specific adaptive immune responses. Interestingly, some of these CSGs were detected in ileum and cecum, sites in which free-living populations of the pathogen reside at considerably lower biomasses than populations in the colon. These microbial signatures, which occur proximal to the primary site of infection, could reflect direct interactions with the pathogen, but given the lower biomasses of pathogen at these sites, may well reflect effects of systemic host responses elicited in response to the colitis. Finally, MC-TIMME detected CSGs exhibiting prolonged increases after clearance of the pathogen, such as CSGs represented by the genus *Clostridium* (OTU#24) in ileum or cecum. During the recovery and convalescent phases, the host resolves local inflammatory responses and actively repairs damaged tissues, a process that takes weeks to complete. The finding that some organisms attain higher biomass during this phase suggests that these taxa may promote or otherwise benefit from changes in intestinal microenvironments that occur during tissue repair and resolution of inflammation, a pattern warranting further evaluation in other models of colitis.

Consensus Signature Groups provide a useful method to compress the potentially enormous variety of dynamic behaviors seen across taxa into smaller sets of interpretable patterns. Many CSGs contained phylogenetically diverse taxa, identifying groups of organisms that may share functional traits needed to compete with the pathogen or otherwise take advantage of microenvironments changing in concert with evolving host responses. These CSGs identify candidate taxa and species to validate in further directed experimental systems. Conversely, CSG analysis also found closely related taxa with dramatically different behaviors during infection, such as members of the families Clostridiaceae and Lactobacillaceae in cecum and ileum. In particular, MC-TIMME used sequencing data to discover three *Lactobacillus* species with different dynamic behaviors, which were subsequently validated with quantitative culture data. Of note, prior studies in *C. rodentium* and other mouse models of colitis have suggested that these three *Lactobacillus* species may behave differently in specific intestinal microenvironments, exerting different effects on the host [38], [39].

Analyses on species detectable only with culture also demonstrated significant effects of *C. rodentium* infection on the dynamics of related *Enterobacteriacaea*, namely mouse commensal species of *P. vulgaris* and *E. hormachei*. In the cecum of infected mice, *E. hormachei's* trajectory generally paralleled that of *C. rodentium*. *Enterobacter* species are the most similar phylogenetically and metabolically to *Citrobacter* species [40]. These findings would thus suggest that, rather than directly competing for nutrients, *C. rodentium*'s introduction into the ecosystem results in conditions also favorable for *E. hormachei*, with negligible or negative impact upon *P. vulgaris*. The mechanism(s) underlying these interactions are likely complex, but subject to experimental analysis in *in vitro* co-culture systems and specific association studies in germfree mice, and may include direct interactions among species, as well as host-elicited alterations to the gut luminal environment, stimulated by active infection and necessary immune and epithelial responses that ultimately clear the pathogen from enteric environments.

Our computational model provides a general framework for analyzing data from longitudinal studies of the microbiota. In this study, we used MC-TIMME to analyze 16S rDNA phylotyping and quantitative culture data. The model is also applicable to other time-series of counts, including longitudinal sequence-based metagenomic and transcriptomic data. The model's ability to automatically discover and compress a potentially large range of dynamic patterns into smaller and refined sets holds value for extracting relevant signatures from rich time-series datasets. Further, because MC-TIMME uses a fully specified probabilistic model, additional covariates may be readily incorporated into the analysis, which will be essential for supporting future studies linking dynamic changes in the human microbiota to patient phenotypes and outcomes from disease.

## Methods

### *C. rodentium* infection and tissue harvest

This study was carried out in strict accordance with the recommendations in the Guide for the Care and Use of Laboratory Animals of the National Institutes of Health. The protocol was approved by the Institutional Animal Care and Use Committee (IACUC) for Brigham & Women's Hospital (Permit Number: A-3431-01). All efforts were made to minimize suffering. Male C57BL/6 mice at 3 weeks of age were purchased from Taconic Farms (Taconic, NY) and maintained in-house for 2 weeks prior to challenge at 5 weeks of age with 5 X 10^7^ CFU/mouse of strain DBS100 of *C. rodentium* as described [23], [41]. Prior to inoculation, bedding was mixed and distributed among cages twice a week to limit development of varied flora across mice. Post-inoculation, longitudinal tissue samples were harvested at the same time each day. For tissue collection, mice were placed in pre-sterilized plastic containers for up to 30 minutes to allow clearance of fecal pellets from the distal colon through normal defecation. Mice were anesthetized and sacrificed by overdose with volatile isoflurane (Vedco Inc., St. Joseph, MO). The abdomen was sprayed with 70% ethanol to wet the fur. To prevent cross-contamination of intestinal contents across samples harvested from gut tissues, three sets of sterile, UV-irradiated tools were used on each mouse to open skin, the abdominal cavity, and then to remove the digestive tract *en bloc*. Individually sterilized razor blades were used on each tissue location to remove 1.0 cm segments of ileum, 2–5 cm proximal to the ileo-cecal valve, 0.5 cm of cecum taken 0.5 cm proximal from the cecal tip, and terminal 3 cm of distal colon, starting 0.5 cm from the anal canal. Segments of distal colon were harvested that lacked fecal pellets, to insure detection of resident colonic flora.

Ileum, cecum and distal colon (devoid of fecal pellets) from infected mice and uninfected controls were harvested at days 3, 7, 10, 14, 21 and 28 post-inoculation for 16S rDNA gene sequencing and quantitative culture of the pathogen and predominant commensals. Samples to be subjected to 16S rDNA gene sequencing were snap frozen on liquid nitrogen and stored at −80°C until processed for DNA. Samples to be used for culturing were placed in 1.0 mL of pre-reduced phosphate buffered saline (PBS) containing 40 mM L-cysteine-HCl (Sigma Chemical, St. Louis, MO). Remaining segments were placed in 10% zinc-buffered formalin for fixation and paraffin-embedding to evaluate tissue histopathology.

### Quantitative aerobic and anaerobic culture

Preliminary cultures, conducted prior to infection, were used to identify dominant aerotolerant and obligately anaerobic commensals (data not shown). In particular, efforts were directed at 1) commensal species demonstrating counts of 1 X 10^8^ CFU/gram or higher in cecum, and preferably in ileum or distal colon, with the intent of providing “reference” species that could be detected both by culture and pyrosequencing, and 2) members of the Enterobacteriaceae to ascertain if the 16S rDNA V1 and V2 gene regions could discriminate enteric commensals from *C. rodentium*. From this initial screen, species of *Lactobacillus* and were found to be the most prevalent, culturable commensals with biomass >10^8^ CFU/g of tissue. Though members of the *Bacteroidales* and *Clostridiales* were also cultured, these species did not exceed this threshold.

Samples were weighed prior to homogenization in an anaerobic Coy chamber with serial dilution and plating to the media described in Table 2. Quantitative culture was used to obtain species-level counts for the pathogen, *Citrobacter rodentium*, and for the commensals listed in Table 2. With the exception of MacConkey agar with 10 µg/mL of Tetracycline (Sigma Chemical, St. Louis, MO) all agar media listed in Table 2 were commercially purchased from Remel (Lenexa, KS). Aerobic incubation was in a 5% CO_2_ humidified incubator at 37°C. Colony types were enumerated at 24 and 48 hours of incubation. Anaerobic incubation was conducted in a Coy chamber with atmosphere of 10% carbon dioxide, 10% hydrogen and 80% nitrogen at 37°C. Plates were incubated for a minimum of 72 hours after which colony types were enumerated and described. Representative isolates from each tissue sample were re-streaked to anaerobic Brucells modified broth (BMB) agar, Gram stained, and aerotolerance tests were performed to confirm obligate anaerobes versus aerotolerant species.

***Citrobacter rodentium*** was identified by growth on MacConkey agar as dark pink, lactose-positive, indole-negative colonies, producing Gram-negative rods by morphology. Representative isolates were typed by API-20E panels (Biomérieux, Durham, NC) and full 16S rDNA sequencing.

***Enterobacter hormachei*** was identified as opaque, light pink, lactose-fermenting, Gram-negative rods on MacConkey agar, and typed by API-20E panel and full 16S rDNA sequence. This species produced larger colonies than *C. rodentium* at 24 hours of growth and thus was distinguishable within dense growth of the pathogen. Putative isolates of *E. hormachei* from acutely infected mice were selected and re-streaked to fresh MAC plates to verify the species.

***Lactobacillus johnsonii*** was identified by growth on CNA agar, producing slender, elongated and non-sporulating Gram-positive rods that were catalase negative. Isolates were susceptible to vancomycin. Full 16S rDNA sequence was used to speciate representative isolates from each time point.

***Lactobacillus murinus*** was identified by growth on CNA and BKV agar (vancomycin-resistant) that produced non-sporulating Gram-positive rods that were catalase negative. Isolates grew on BKV containing vancomycin. Full 16S rDNA sequence was used to speciate representative isolates from each time point.

***Lactobacillus reuteri*** was identified by preferential growth under anaerobic conditions on BMB and CNA agar. Cell morphology by Gram stain showed shorter Gram-positive, non-spore forming rods that were catalase-negative. Full 16S rDNA sequence was used to speciate representative isolates from each time point.

***Proteus vulgaris*** was the only organism to grow on MacConkey agar + 10 µl/mL tetracycline where it produced pale, lactose-negative colonies. Identification was confirmed by API-20E typing of representative isolates from each time point, and by full 16S rDNA sequence.

After final counts of each species at the highest dilution at which it was detected, counts were entered into a spreadsheet containing the starting tissue mass and dilution factor to obtain log_10_ of colony forming units per gram of input tissue mass (log_10_ CFU/g).

### 454 Pyrosequencing of 16S rDNA gene signatures

DNA was extracted from mouse fecal pellets using the MoBio Fecal DNA extraction kit per the manufacturer's specifications (MoBio Laboratories Inc, Carlsbad, CA). Fragments of the 16S rRNA gene, spanning the V1 and V2 hypervariable regions were PCR amplified from each tissue sample using sample-specific barcodes adapted to universal 16S rDNA primers 27F (5′-AGAGTTTGATCMTGGCTCAG-3′) and 338R (5′-GACTCCTACGGGAGGCWGCAG-3′). DNA was sequenced using a Genome Sequencer FLX and GS-LR70 kit (Roche Applied Sciences, Indiannapolis, IN) at Duke University's IGSP Sequencing Core Facility (Durham, NC). Raw sequence datasets have been deposited at NCBI's Short Read Archive (SRA) under BioProjectID PRJNA202962.

### Data preprocessing

Mothur v.1.14.0 was used to preprocess sequences, and to construct and taxonomically classify OTUs [4]. The pipeline used was as follows:

1. Barcodes were trimmed and sequences were filtered based on quality scores with the following parameters used: window average quality score ≥ 35, window size  = 50, no ambiguous bases, homopolymer length ≤ 8, primer differences ≤ 2, barcode differences ≤ 1, and length between 200 bp and 300 bp.
2. Sequences were then chopped to 200 bp and aligned against the SILVA compatible database provided with mothur. Sequences that started before the 2.5-percentile or ended after the 97.5-percentile in the alignment were filtered out.
3. The ChimeraSlayer and preclustering algorithms implemented in mothur were run.

Table 4 indicates the number of reads after each filtering step.

**Table 4:** Filtering steps in bioinformatics pipeline and remaining sequencing reads.

Filtering step	Sequences remaining after filter
None	678,247
QC scores, ambiguous bases, homopolymers, min/max length, match barcodes	300,462
Alignment to 16S rDNA reference database	294,996
ChimeraSlayer	274,882

Sequences were assigned to OTUs using the furthest neighbor method and a threshold of 97% similarity. Distances between sequences were calculated in mothur, and sequences were assigned to OTUs using the furthest neighbor method with a threshold of 97% similarity. **OTU numbers were assigned by the mothur pipeline, which orders OTUs in descending order by the sum of the sequencing reads across all samples assigned to the OTU**. The consensus taxonomic classification for each OTU was determined using the naïve Bayes classifier [33] implemented in mothur against the RDP database (bootstrap cutoff of 60%).

After filtering OTUs that failed to have ≥5 reads in ≥3 samples, 210 OTUs were available for input to MC-TIMME. Relationships among the 210 OTUs were visualized by building a tree of the most abundant sequence from each OTU and rendering the results using the Dendroscope software [42]. The tree was constructed using mothur, by aligning sequences against the included ARB SILVA reference database, calculating distances between sequences using the dist.seqs command (default options), and then building a neighbor joining tree with the clearcut command (default options).

### Sequencing count normalization

To make sequence counts for OTUs comparable across samples, we used a nonparametric regression method, Locally Weighted Scatterplot Smoothing (LOWESS). This technique allows for nonlinear normalization, which has been shown to be important for data in which error characteristics differ substantially at the extreme ranges of values such as with DNA microarray data [43]. This error characteristic is evident in our data, as shown in Figure S1. The LOWESS normalization on our data compensates for differing numbers of sequencing reads in samples, with allowance for a nonlinear relationship for samples with very low or high numbers of reads.

To perform the LOWESS regression, all OTU counts across all samples were included in the regression. The independent variable for the regression was the relative abundance of the OTU in the sample, and the dependent variable was the counts for the OTU in the sample. We used the MATLAB 2010b (MathWorks, Natick, MA) malowess function with the robust regression option and with second degree curves to perform the analysis. Counts were then rescaled and rounded based on the LOWESS output. These normalized counts were then used as input for the MC-TIMME algorithm (Dataset S1).

### Microbial Counts Trajectories Infinite Mixture Model Engine (MC-TIMME)

MC-TIMME is based on Dirichlet Process or Infinite Mixture Models, a class of nonparametric Bayesian models in which data is assumed to be generated from a weighted mixture of a potentially unlimited number of components [29]. Mixture components correspond to prototype signatures, with component *k* defined by a set of signature shape variables δ*_kt_* and a variable controlling data variance ε*_k_*. Observed count data is modeled using a Generalized Linear Model (GLM) with a Negative Binomial Distribution (NDB) data model with mean dependent on δ*_kt_* and variance controlled by ε*_k_*. The model also specifies prior probability distributions on model parameters controlled by hyperparameters. We extended the original MC-TIMME model to use a continuous-time model of dynamics based on a Gaussian random with time-scaled variance (Protocol S1). We derived an efficient Markov Chain Monte Carlo (MCMC) approximate inference algorithm with Gibbs sampling steps for assignments of time-series of counts for OTUs to prototype signatures, and specialized Metropolis-Hastings (MH) steps for trajectory variable and hyperparameter updates. We used 25,000 MCMC iterations for burn-in and then an additional 75,000 iterations to estimate the posterior distribution.

### Detecting signature changes in response to infection

Our model is fully Bayesian and thus does not use *p*-values. We instead directly estimate probabilities of signatures differing (Protocol S1). We used the following criteria to detect changes in OTU signatures in response to introduction of the pathogen: a) the posterior probability of infected and uninfected samples sharing a prototype signature was <5%, and b) the 95% credible intervals for infected and uninfected signatures did not overlap for at least two time-points. Using these criteria, we estimated the false rate of detection at <5% across our dataset.

### Correlation analyses of sequence and culture-based trajectories:

To assess the similarity of culture and sequence-derived signatures, we performed the following analysis. For the culture-derived signatures, we computed the difference between signatures in the infected and uninfected states for each organism in each tissue site. We performed analogous computations for all sequence-derived OTU signatures (including for OTUs for which there was no corresponding culture data). Then, for each differenced culture-derived signature, we computed its uncentered correlation coefficient [44] against all the differenced sequence-derived signatures. This analysis provided an indication of how similar or different a given culture signature was from all OTU signatures analyzed in the longitudinal dataset, including the OTU signature associated with the given isolate cultured. We then computed the percentage of these correlations that were less than the actual correlation between the culture-derived differenced signature and the corresponding sequence-derived differenced signature. For the computation of this percentage, we excluded OTUs classified to the same genus as the cultured organism being evaluated.

## Supporting Information

Figure S1 **Locally Weighted Scatterplot Smoothing (LOWESS) normalization of sequence count data.** The LOWESS non-parametric regression method was applied to sequencing data to normalize counts obtained across samples. Data (blue plus signs) are plotted with the relative abundance as the independent variable and sequence counts as the dependent variable. Each data point represents an Operational Taxonomic Unit (OUT) from a single sample (a particular time-point, tissue and replicate). The red line indicates the best linear fit to the data. Deviations from the linear fit are evident at the lower and upper range of data values. The black dots represent the fitted values from the LOWESS regression. The LOWESS estimated values were rounded down and used as the effective number of reads for each OTU.
(TIF)

Protocol S1 **Detailed description of the MC-TIMME algorithm and associated computational methods.**
(PDF)

Table S1 **Sample key.**
(TXT)

Table S2 **Sample barcode key for samples submitted for 16S rRNA gene sequencing.**
(TXT)

Dataset S1 **LOWESS normalized counts for Operational Taxonomic Units from 16S rDNA phylotyping data.**
(TXT)

Dataset S2 **Colony Forming Units per gram of input tissue for cultured organisms.**
(TXT)
