# Supplementary figures and images for: Dynamics of the Microbiota in Response to Host Infection
Source: PLoS One. 2014 Jul 11;9(7):e95534. doi: 10.1371/journal.pone.0095534 (PMC4094490; doi:10.1371/journal.pone.0095534)

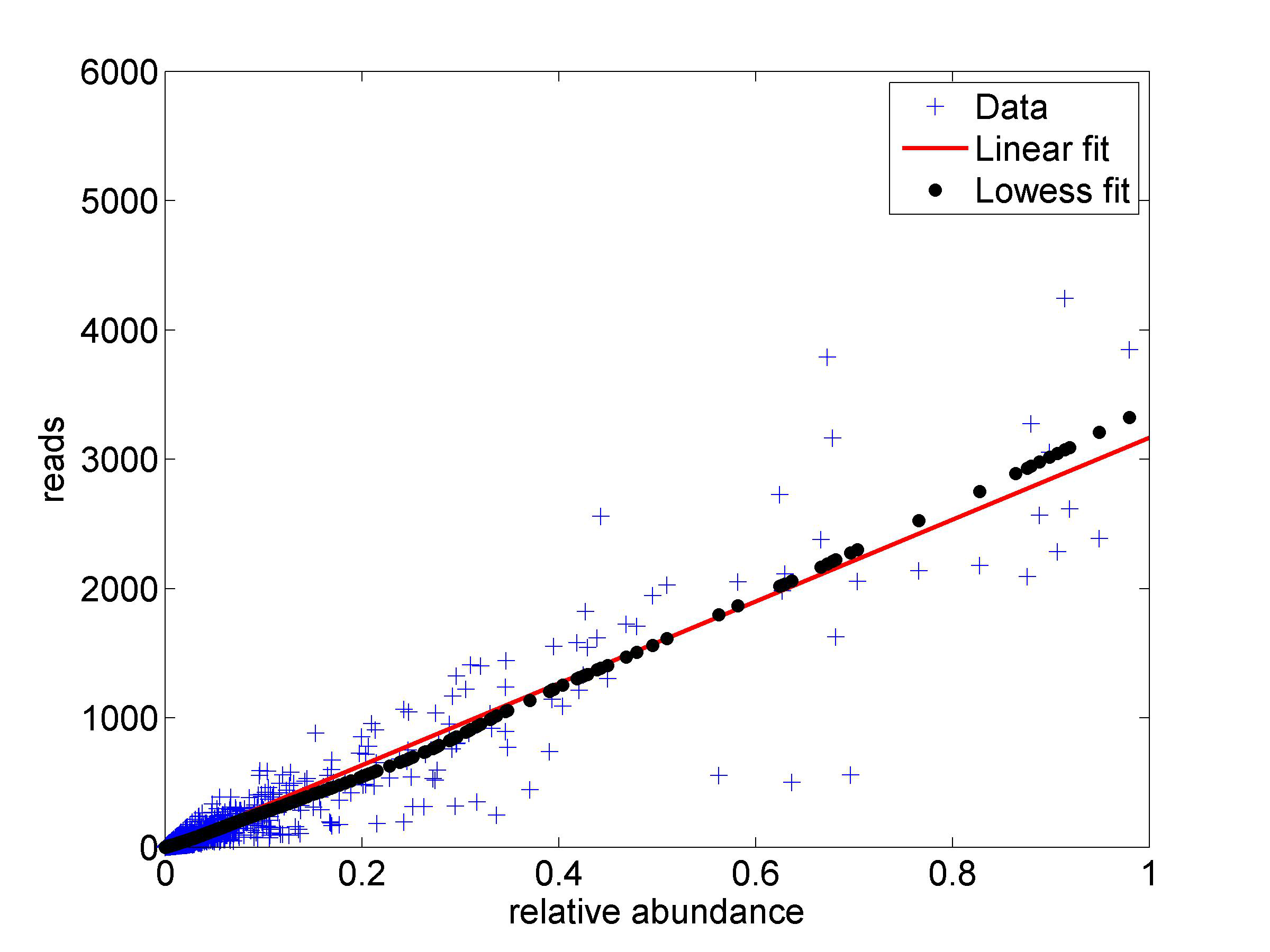

Supplement: Figure S1 — Locally Weighted Scatterplot Smoothing (LOWESS) normalization of sequence count data. The LOWESS non-parametric regression method was applied to sequencing data to normalize counts obtained across samples. Data (blue plus signs) are plotted with the relative abundance as the independent variable and sequence counts as the dependent variable. Each data point represents an Operational Taxonomic Unit (OUT) from a single sample (a particular time-point, tissue and replicate). The red line indicates the best linear fit to the data. Deviations from the linear fit are evident at the lower and upper range of data values. The black dots represent the fitted values from the LOWESS regression. The LOWESS estimated values were rounded down and used as the effective number of reads for each OTU. (TIF) [file pone.0095534.s001.tif]
