# Supplementary material for: Dynamics of the Microbiota in Response to Host Infection
Source: PLoS One. 2014 Jul 11;9(7):e95534. doi: 10.1371/journal.pone.0095534 (PMC4094490; doi:10.1371/journal.pone.0095534)
Supplement: Protocol S1 — Detailed description of the MC-TIMME algorithm and associated computational methods. (PDF) [file pone.0095534.s002.pdf]

Protocol S1: Detailed description of the MC-TIMME algorithm and associated computational methods

1 Overview

The original MC-TIMME model is described in [1]. For completeness, in this appendix we describe the original core model as well as the extensions made to the model in the present work.

MC-TIMME is based on Dirichlet Process or Infinite Mixture Models, which are a class of nonparametric Bayesian models. A Finite Mixture Model (FMM) assumes that data $\mathbf{y}$ arise from a weighted mixture of K probability distributions:

$$\mathrm{p}(\mathbf{y}) = \sum_{k=1}^{K} \pi_k \mathrm{f}(\boldsymbol{y};\ \boldsymbol{\theta}_k)$$

Here, $\boldsymbol{\pi}$ are mixture weights (probabilities) such that $\sum_{k=1}^{K} \pi_k = 1$, $\boldsymbol{\theta}_k$ are the parameters for mixture component k, and $\mathrm{f}(\cdot;\ \cdot)$ is a probability density function for the mixture components.

An Infinite Mixture Model (IMM) is a nonparametric extension of the FMM, in which the number of components is potentially unlimited and varies with data. The IMM is fully Bayesian, with prior distributions placed on each model variable.

For the MC-TIMME model, the mixture components are the prototype signatures. Each prototype signature is characterized by a model of dynamics, described by a set of variables that specify the signature shape and the variance of data points around the signature. Different models of dynamics can be "plugged" into MC-TIMME to capture different perturbations to the microbiota. In the present work, we extend the original MC-TIMME model to capture perturbations to the microbiota induced by an ongoing enteric infection in the host.

The unit of observation is a set of counts measured over time, e.g., counts of sequences for an organism in a particular condition. MC-TIMME models latent (hidden) variables that assign the observed sets of counts to prototype signatures. The number of prototype signatures, their shapes, and other model variables are unknown and inferred from the data.

MC-TIMME has several features specifically designed to leverage potentially sparse and noisy time-series count data. First, by assigning taxa to discrete prototype signatures, MC-TIMME strengthens statistical inferences by pooling taxa with similar dynamics to estimate shared model parameters such as signature shapes and data variances. Second, MC-TIMME uses a Negative Binomial Distribution error model, which has been shown to outperform other models for count data from high-throughput sequencing [2, 3] and microbial culture experiments [4]. Third, MC-TIMME explicitly models dependence among time-points, which aids in estimating signature values in the setting of noise or low numbers of replicates. Finally, MC-TIMME simultaneously analyses the entire time-series and all taxa to increase statistical power. As a

consequence of this combination of techniques, in the present analysis, MC-TIMME can effectively detect changes between infected and uninfected populations for cases in which the number of replicates at each time-point is small, which simple statistical methods cannot.

2 Comments on Bayesian methods and mixture models

Unlike classical frequentist approaches that regard model parameters as fixed, Bayesian approaches regard model parameters as variables subject to error and thus governed by probability distributions. This leads to different inference tasks for frequentist and Bayesian approaches.

For a frequentist approach, the inference task is to calculate point estimates $\widehat{\boldsymbol{\theta}}$ of the unknown model parameters $\boldsymbol{\theta}$ based on the observed data $\mathbf{y}$. One then calculates a p-value, which is the probability of obtaining the observed value $\widehat{\boldsymbol{\theta}}$ or one more extreme from a null model (i.e., a model with pre-specified parameter settings $\boldsymbol{\theta}_0$).

In contrast, the Bayesian inference task is to estimate the posterior probability distribution, $\mathrm{p}(\boldsymbol{\theta} \mid \mathbf{y})$, which is the distribution of model variables $\boldsymbol{\theta}$ given all observed data $\mathbf{y}$. Using Bayes' rule, this can be written in terms of the data likelihood $\mathrm{p}(\mathbf{y} \mid \boldsymbol{\theta})$ and the prior probability distribution of model parameters $\mathrm{p}(\boldsymbol{\theta})$:

$$\mathrm{p}(\boldsymbol{\theta} \mid \mathbf{y}) = \frac{\mathrm{p}(\mathbf{y} \mid \boldsymbol{\theta}) \cdot \mathrm{p}(\boldsymbol{\theta})}{\mathrm{p}(\mathbf{y})}$$

Because in the Bayesian setting $\boldsymbol{\theta}$ is assumed to have a probability distribution, one can directly calculate posterior probabilities of interest regarding model parameters. For instance, in a clinical trial of an antihypertensive drug, one might be interested in the posterior probability that systolic blood pressure decreases by some prespecified percentage in subjects. In the present application of MC-TIMME, we are interested in the posterior probability that time-series of counts from different taxa (or the same taxon under different conditions) are assigned to a prototype signature.

A classic problem in statistics and signal processing is the detection of signals in noisy data. A common approach is to model the noise only, and assume the null hypothesis that a given piece of data (e.g., an observed time-series) comes from this noise distribution. One then computes a p-value and rejects the null hypothesis if the p-value is less than some threshold value. An inherent limitation of p-values is that they only quantify the strength of evidence against the null model, but do not provide a direct measure of confidence that data arise from an alternate model [5].

Mixture Models provide a natural framework for incorporating multiple hypotheses, and thus for computing statistics that provide information about multiple hypotheses simultaneously. Bayesian Mixture Models are particularly useful in this context, as they allow one to incorporate prior knowledge and compute distributions of mixture parameters. Consider a simple example of a two component Bayesian Mixture Model, in which the two components and their weights are prespecified. To make the example more concrete, one can imagine that the components represent two distinct populations (e.g., microbes exhibiting two different types of growth signatures) or signal and noise components. Assume that $\mathbf{y}_i$ is a time-series of counts, and let z_i denote a latent variable indicting to which component $\mathbf{y}_i$ belongs. Then, the posterior probability that $\mathbf{y}_i$ belongs to component 2 is given by:

$$\mathrm{p}(\mathsf{z}_i = 2 \mid \pi_1, \pi_2, \theta_1, \theta_2, \boldsymbol{y}_i) = \frac{\pi_2 \mathrm{f}(\boldsymbol{y}_i;\ \theta_2)}{\pi_1 \mathrm{f}(\boldsymbol{y}_i;\ \theta_1) + \pi_2 \mathrm{f}(\boldsymbol{y}_i;\ \theta_2)}$$

Thus, we see from this equation that the posterior probability incorporates information about both model components.

Bayesian Mixture Models can also inherently "adjust for multiple hypotheses" (see [6]). To see this, consider again a simple two component Mixture Model, but now consider a model in which the mixing parameters $\boldsymbol{\pi}$ are regarded as random variables with an appropriate prior distribution. Assume that we have observed a large number of microbial time-series, and that most of these time-series belong to component 1 (e.g., the "noise" component). This means that the mixture weight π_1 for component 1 will have a posterior probability distribution centered close to one and π_2 will have a posterior probability distribution centered close to zero. Thus, if we observe a new microbial time-series $\mathbf{y}_i$ that truly should belong to component 2 (e.g., is a true "signal"), a large π_1 "bias" will need to be overcome tending to assign $\mathbf{y}_i$ to component 1 (e.g., tending to identify the time-series as noise). This effect will only be overcome if $\mathbf{y}_i$ has a very good fit to the component 2 probability density $\mathrm{f}(\boldsymbol{y}_i;\ \theta_2)$ (e.g., the time-series really looks like a "signal"). Note that the posterior probability distributions for π_1 and π_2 depend on *all* the data, and so inherently account for multiple hypotheses. In this sense, Bayesian Mixture Models automatically control for false assignments or detections. This argument can be extended to Bayesian Mixtute Models with multiple components, including Infinite Mixture Models such as MC-TIMME. We tested this feature of our model empirically using repeated random permutations of our dataset, and estimated a false detection rate across the entire dataset of less than 5%, indicating that our model behaved as we theoretically predicted. See Section 7 for details.

3 Model definition

Figure 1 depicts the extended MC-TIMME model structure in graphical model form with plate notation. The model is hierarchical, with variables at higher levels parameterizing prior distributions for variables at lower levels. We assume that we have observed counts (e.g., sequencing reads, colony forming units, etc.) for G taxa in S sites (e.g., body tissues) in I conditions (e.g., infected and control subjects) at T time-points. We further assume we have $R(g, s, i, t)$ replicates for a given experiment, which may vary as a function of the taxa, site, condition and time-point.

We assume that there are a potentially infinite number of prototype signatures with mixture weights $\boldsymbol{\pi}$ and mixture parameters $\boldsymbol{\Theta}$. Each taxon g in site s and condition i is assigned to a prototype signature via a variable $\mathsf{z}_{gsi} \mid \boldsymbol{\pi} \sim \text{Multinomial}(\boldsymbol{\pi})$.

To extend MC-TIMME for modeling the microbiota in a host with an ongoing infection, we use the following continuous-time model of dynamics. For a given prototype signature k, the parameter vector $\boldsymbol{\theta}_k$ consists of signature shape variables δ_{kt} and a variable ϵ_k controlling data variance:

$$\boldsymbol{\theta}_k = (\delta_{k1}, \ldots, \delta_{kT}, \epsilon_k)$$

Here, δ_{k1} corresponds to the starting value for the signature k. To capture temporal dependencies, we assume that the value of the signature at time t, $\eta_k(t)$, is generated via a random walk over the δ_{kt} variables:

$$\eta_k(t) = \sum_{1 \leq \tau \leq t} \delta_{k\tau} \tag{1}$$

The data generation model is then as follows. Let y_{gsitr} represent the observed counts for taxon g in site s in condition i at time-point t and for replicate r. As in the original MC-TIMME model [1], we assume

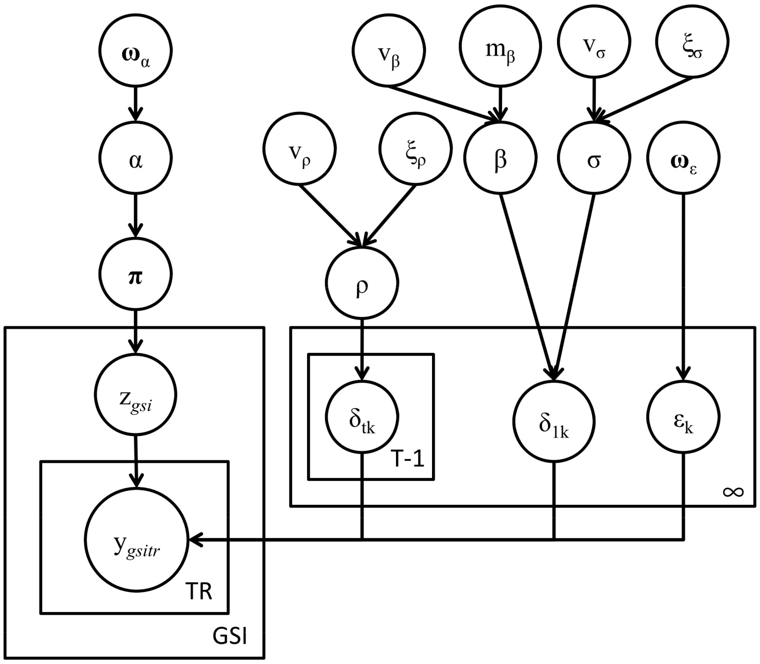

Figure 1: The MC-TIMME model depicted in graphical model form. Circles represent random variables and edges represent dependencies. Rectangles (plates) represent repeated variables. For example, the variable y_{gsitr} represents the observed counts for taxon g in site s in condition i at time-point t and for replicate r. This variable lies within two overlapping plates, one of which represents repetition over the G taxa, S sites and I experimental conditions, and the other of which represents repetition over the T time-points and R replicates. Arrows going into y_{gsitr} indicate that this variable depends directly on several others. The variable z_{gsi} represents a probabilistic assignment of taxon g in site s in condition i to a particular prototype signature. The variables δ_{k1} and δ_{kt} specify the shape of prototype signature k, and ϵ_k controls the variability of data generated by the signature. The infinity sign in the corner of the plate surrounding these variables indicates that there are a potentially unlimited number of prototype signatures. Thus, the value of an observed data point y_{gsitr} depends on its probabilistic assignment to a prototype signature and the corresponding shape and variability parameters for the signature. Variables higher in the hierarchy depict prior distributions for child variables, or hyperparameters for the prior distributions.

that the conditional distribution for the observation y_{gsitr} is the Negative Binomial Distribution (NBD), parameterized by the exponentiated random walk of δ_k variables and a variable ϵ_k controlling variance of the data for prototype signature k:

$$\mathsf{y}_{gsitr} \mid \mathsf{z}_{gsi} = k, \boldsymbol{\theta}_k \sim \text{Neg-Bin}(\mu_k(t), \epsilon_k) \tag{2}$$

$$\mu_k(t) = exp(\eta_k(t)) \tag{3}$$

$$\text{Neg-Bin}(y_{gsitr}; \mu_k(t), \epsilon_k) = \frac{\Gamma(\mathsf{y}_{gsitr} + \epsilon_k)}{\Gamma(\epsilon_k)\mathsf{y}_{gsitr}!} \cdot \frac{\mu_k(t)^{\mathsf{y}_{gsitr}} \epsilon_k^{\epsilon_k}}{(\mu_k(t) + \epsilon_k)^{\mathsf{y}_{gsitr} + \epsilon_k}}$$

Thus, the data is assumed to be generated via a Generalized Linear Model (GLM) using the Negative Bionimal Distribution, with parameterization dependent on the prototype signature to which the data is assigned.

To complete the model, priors are placed on variables as follows. We assume that the δ_{kt} variables specifying signature values have Normal priors. For the starting value of the signature δ_{k1}, we assume a Normal prior with mean β and variance σ^2:

$$\delta_{k1} \mid \beta, \sigma \sim \text{Normal}(\beta, \sigma)$$

For subsequent signature values δ_{kt} for $t > 1$, we assume a Normal prior with mean zero and time-varying variance $\rho^2 \Delta t$:

$$\delta_{kt} \mid \rho \sim \text{Normal}(0, \rho\sqrt{\Delta t}) \quad \text{for } 2 \leq t \leq T$$

Here, Δt is the interval between time-point t and the previous time-point. Thus, the variance of the random walk increases with larger time intervals.

For β, the mean of the δ_{k1} signature starting value, we also assume a Normal prior $\beta \sim \text{Normal}(m_\beta, v_\beta)$.

For σ^2, the variance of δ_{k1}, we assume a conjugate scaled inverse-chi-square prior:

$$\sigma^2 \mid v_\sigma, \xi_\sigma \sim \text{Inv-}\chi^2(v_\sigma, \xi_\sigma)$$

$$\text{Inv-}\chi^2(\sigma^2; v_\sigma, \xi_\sigma) = \text{Inv-Gamma}(\sigma^2; \xi_\sigma/2, \xi_\sigma v_\sigma/2)$$

For ρ^2, the random walk variance, we also assume a conjugate scaled inverse-chi-square prior:

$$\rho^2 \mid v_\rho, \xi_\rho \sim \text{Inv-}\chi^2(v_\rho, \xi_\rho)$$

For ϵ_k, the parameter controlling variance for the NBD for component k, we assume a Gamma prior, $\epsilon_k \mid \boldsymbol{\omega_\epsilon} \sim \text{Gamma}(\boldsymbol{\omega_\epsilon})$.

The mixture weights $\boldsymbol{\pi}$ are assumed to have a stick-breaking prior, $\boldsymbol{\pi} \sim \text{Stick}(\alpha)$, where α is a concentration parameter. The stick-breaking prior generates a countably infinite partition of the unit interval [0,1], with the "evenness" of the partition controlled by the concentration parameter α. The stick-breaking prior on $\boldsymbol{\pi}$ is defined constructively as:

$$\pi'_j \mid \alpha \sim \text{Beta}(1, \alpha)$$

$$\pi_j = \pi'_j \prod_{l=1}^{j-1} (1 - \pi'_l)$$

See [7] for further details regarding the stick-breaking prior formulation for the Dirichlet Process.

Finally, we assume that α has a Gamma prior, $\alpha \mid \boldsymbol{\omega_\alpha} \sim \text{Gamma}(\boldsymbol{\omega_\alpha})$.

4 Further modifications for handling quantitative culture data

Culture data yields information on not only observed counts of Colony Forming Units (CFUs), but also provides the tissue mass assayed and the dilution factor of the sample. We incorporate this information as a per sample offset term in the model. To be precise, let y_{gsitr} be the CFUs for organism g in site s in condition i at time t, let weight_{gsitr} be the weight of the tissue sample in grams, and let dil_{gsitr} be the dilution factor. The offset term o_{gsitr} is then calculated as:

$$o_{gsitr} = \frac{1}{\text{weight}_{gsitr} \cdot \text{dil}_{gsitr}}$$

$$\overline{o} = \underset{g,i,t,r}{\text{median}}\, o_{gsitr}$$

The conditional probability in Equation 2 for y_{gsitr}, the observed CFU counts, is then modified to be:

$$\mathsf{y}_{gsitr} \mid \mathsf{z}_{gsi} = k, \boldsymbol{\theta}_k \sim \text{Neg-Bin}(\mu_k(t) \cdot o_{gsitr}/\overline{o}, \epsilon_k) \tag{4}$$

5 Hyperparameter settings

We set the means of prior distributions empirically from the data and we set the variances of prior distributions to relatively large values to create diffuse or "uninformative priors." We performed sensitivity analysis on hyperparameters and found that our results were relatively insensitive to the particular settings used (see below).

For the concentration parameter $\alpha \mid \boldsymbol{\omega_\alpha} \sim \text{Gamma}(\boldsymbol{\omega_\alpha})$, we set its hyperparameters to weakly favor assigning all data to a single signature (and thus biasing toward no change in infected versus uninfected samples). It can be shown that the expected number of non-empty components K for a dataset with N data points is given by [8]:

$$\text{E}[K \mid \alpha, N] = \alpha \ln \sum_{l=K-1}^{N} \frac{1}{\alpha + l - 1} \approx \alpha \ln \frac{N+\alpha}{\alpha}$$

In this context, N is the total number of observed time-series of counts, i.e., $N = G \cdot I$, where G is the number of taxa and $I = 2$ (accounting for both infected subjects and uninfected controls).

We thus set the expectation equal to one and solve for $\widehat{\alpha}$:

$$\widehat{\alpha} \ln \frac{N + \widehat{\alpha}}{\widehat{\alpha}} = 1$$

We then set $\omega_{\alpha 1} = \widehat{\alpha}$. We set $\omega_{\alpha 2} = 0.5$; we tested a 5-fold range of values for $\omega_{\alpha 2}$ around the value used in the manuscript, and found that our results were insensitive to the parameter setting.

For $\epsilon_k \mid \boldsymbol{\omega_\epsilon} \sim \text{Gamma}(\boldsymbol{\omega_\epsilon})$, the variable controlling the NBD variance for prototype signature k, we set the hyperparameters follows. We first computed the empirical NBD mean $\widetilde{\mu}_{gsit}$ and variance $\widetilde{v}_{gsit}$ using a maximum likelihood estimator for each set of replicates for a given taxon g in site s in condition i and time-point t. The hyperparameter $\omega_{\epsilon 1}$ was then set as:

$$\omega_{\epsilon 1} = \underset{g,i,t}{\text{median}} \left\{ \frac{\widetilde{m}_{gsit}^2}{\widetilde{v}_{gsit} - \widetilde{m}_{gsit}} \right\}$$

The hyperparameter $\omega_{\epsilon 2}$ was set to 0.5; we tested a 5-fold range of values for $\omega_{\epsilon 2}$ around the value used in the manuscript, and found that our results were insensitive to the parameter setting.

For $\beta \mid m_\beta, v_\beta \sim \text{Normal}(m_\beta, v_\beta)$, the variable specifying the mean of the prior on the initial signature values δ_{k1}, we set the hyperparameters as follows. We first computed the empirical NBD mean $\widetilde{\mu}_1$ and variance $\widetilde{v}_1$ for all samples at the first time point. We then set m_β to the transformed mean:

$$m_\beta = \ln \frac{\widetilde{\mu}_1^2}{\sqrt{\widetilde{\mu}_1^2 + \widetilde{v}_1}}$$

We set v_β to a large variance of 10^3; we tested a 5-fold range of values for v_β around the value used in the manuscript, and found that our results were insensitive to the parameter setting.

For $\sigma^2 \mid v_\sigma, \xi_\sigma \sim \text{Inv-}\chi^2(v_\sigma, \xi_\sigma)$, the variable specifying the variance of the initial signature values δ_{k1}, we set the hyperparameters as follows. We set $v_\sigma = v_\beta$. The parameter ξ_σ can be interpreted as a prior sample size that weights the contribution of v_σ on σ^2. We assumed a small prior sample size of $10 \cdot T$ (e.g., 10 OTUs measured across the time-series) to limit the contribution from the prior.

For $\rho^2 \mid v_\rho, \xi_\rho \sim \text{Inv-}\chi^2(v_\rho, \xi_\rho)$, the random walk variance, we set the hyperparameters as follows. For each taxon g in site s, condition i, and time-point t we computed the empirical count ratio $\widetilde{\delta}_{gsit}$:

$$\widetilde{\delta}_{gsit} = \ln \sum_{r=1}^{R(g,s,i,t)} y_{gsitr}/R(g,s,i,t) - \ln \sum_{r=1}^{R(g,s,i,t-1)} y_{gsi(t-1)r}/R(g,s,i,t-1)$$

We then set v_ρ to:

$$v_\rho = \underset{g,i,t}{\text{median}} \frac{\widetilde{\delta}_{gsit}^2}{\Delta t}$$

The parameter ξ_ρ can be interpreted as a prior sample size. We assumed a small prior sample size of 10 to limit the contribution from the prior.

6 Model inference

As discussed in Section 2, the model inference task for MC-TIMME is to esimate the posterior distribution of all model variables given the observed data. Bayesian inference is notoriously computationally intensive, and presents particular challenges for models such as MC-TIMME that capture temporal dependences and use non-conjugate priors. Below, we describe an efficient Markov Chain Monte Carlo (MCMC) algorithm for approximating the posterior distribution of the MC-TIMME model, which is similar to the inference algorithm for the original MC-TIMME model [1], but with some additional steps to handle the extended model of signature dynamics. Our solution uses a combination of Gibbs sampling, Metropolis-Hastings (MH), and auxiliary variable augmentation steps.

The MCMC sampling loop is as follows:

1. Sample z_{gsi}, the assignment of taxon g in site s in condition i to a prototype signature.

2. Sample prototype signature shape variables δ_{kt}.

3. Sample prototype signature variable ϵ_k controlling NBD variance.

4. Sample β and σ^2, the mean and variance for the prior on initial signature values.

5. Sample ρ^2, the variance of signature random walks.

6. Sample α, the concentration parameter.

6.1 Step 1: sampling assignments of taxa to prototype signatures

For the first step in the MCMC loop, we use the Gibbs sampling method outlined in [9]. We sample the assignment z_{gsi} for taxon g in site s in experimental condition i, conditional on all the data and other model variables including assignments of the other taxa. In this step there are two cases: taxon g in site s in condition i is assigned to an existing component k, or it is assigned to a new component. The update equations are then:

$$\mathrm{p}(\mathsf{z}_{gsi} = k \mid \mathbf{z}_{-gsi}, \alpha, \mathbf{\Theta}, \mathbf{y}) \propto \frac{n_k^{-gsi}}{G - 1 + \alpha} \mathrm{p}(\mathbf{y}_{gsi} \mid \boldsymbol{\theta}_k) \quad \text{for } n_k^{-gsi} > 0 \tag{5}$$

$$\mathrm{p}(\mathsf{z}_{gsi} \neq \mathsf{z}_l, \forall\, l \neq gsi \mid \mathbf{z}_{-gsi}, \alpha, \mathbf{\Theta}, \mathbf{y}) \propto \frac{\alpha}{G - 1 + \alpha} \mathrm{p}(\mathbf{y}_{gsi} \mid \boldsymbol{\theta}_*) \tag{6}$$

Here, n_k^{-gsi} is the number of taxa assigned to prototype signature k excluding taxa g in site s in condition i, and $\mathbf{z}_{-gsi}$ is the assignments of all taxa excluding taxa g in site s in condition i. The variable $\boldsymbol{\theta}_*$ represents the parameters for a new component (prototype signature). The data likelihood for $\mathbf{y}_{gsi}$ is given by:

$$\mathrm{p}(\mathbf{y}_{gsi} \mid \boldsymbol{\theta}_k) = \prod_{\tau=1}^{T} \prod_{r=1}^{R(g,s,i,t)} \text{Neg-Bin}(y_{gsitr};\ \mu_k(t), \epsilon_k)$$

The Gibbs sampling procedure thus either assigns the taxa to an existing prototype signature, or generates a new prototype signature. Because the prior distribution for $\boldsymbol{\theta}$ is non-conjugate, it is not possible to analytically integrate out $\boldsymbol{\theta}_*$. Thus, we follow the method outlined by [9], and sample $\boldsymbol{\theta}_*$ from its prior distribution. Because the sample from the prior distribution is unbiased, this method will sample correctly from the underlying posterior distribution.

6.2 Step 2: sampling prototype signature shape variables

For the second step in the MCMC loop, sampling prototype signature shape variables δ_{kt}, we use Metropolis-Hastings steps. As noted, for a particular prototype signature k, data is assumed to be generated via a GLM using the Negative Bionimal Distribution. A general strategy for Bayesian estimation of GLMs is to use Gibbs sampling combined with Adaptive Rejection Sampling, as the conditional distributions of individual regression coefficients are log-concave [10]. However, this method is not efficient for the MC-TIMME model, which incorporates dependence via the random walk assumption; in practice, we obtained very poor mixing using this method. We instead use a strategy that employs Metropolis-Hastings steps [11]. The key to this method is to generate good linear approximations to the actual GLM distribution to use as proposal distributions. It turns out that these approximations are directly connected to Weighted Least Squares (WLS) methods, which are used for maximum likelihood estimates for GLM parameters.

To make the GLM connection explicit, we can write the NBD mean for prototype signature k at time t in terms of canonical GLM parameters as:

$$h(\mu_k(t)) = \eta_k(t) = \boldsymbol{X}_t' \boldsymbol{\delta}_k$$

The NBD variance is given by:

$$b(\mu_k(t)) = \mu_k(t) + \frac{\{\mu_k(t)\}^2}{\epsilon_k}$$

Here, $h(\cdot)$ represents the link function, and $\boldsymbol{X}_t$ is a vector of regression coefficients. We use the natural logarithm function as the link function $h(\cdot)$. For the random walk used in the MC-TIMME model, $X_{tj} = 1$ for $j \leq t$ and $X_{tj} = 0$ otherwise.

The procedure for creating a WLS estimate is as follows. We first create transformed data values $\widetilde{\mathsf{y}}_{gsitr}$ for all data points assigned to prototype signature k:

$$\widetilde{\mathsf{y}}_{gsitr}(\boldsymbol{\delta}_k) = \eta_k(t) + (\mathsf{y}_{gsitr} - \mu_k(t))h'(\mu_k(t)) \tag{7}$$

We then use the transformed data values to generate a diagonal weight matrix $\boldsymbol{W}(\boldsymbol{\delta}_k)$:

$$W_{gsitr}^{-1}(\boldsymbol{\delta}_k) = b'(\mu_k(t))\{h'(\mu_k(t))\}^2 \tag{8}$$

The moments for the proposal distribution we will use in the MH steps are then given by:

$$\boldsymbol{m}_k^{(j)} = \{\boldsymbol{\Lambda}^{-1} + \boldsymbol{X}'\boldsymbol{W}(\boldsymbol{\delta}_k^{(j-1)})\boldsymbol{X}\}\{\boldsymbol{\Lambda}^{-1}\boldsymbol{a} + \boldsymbol{X}'\boldsymbol{W}(\boldsymbol{\delta}_k^{(j-1)})\widetilde{\mathbf{y}}(\boldsymbol{\delta}_k^{(j-1)})\} \tag{9}$$

$$\boldsymbol{C}_k^{(j)} = \{\boldsymbol{\Lambda}^{-1} + \boldsymbol{X}'\boldsymbol{W}(\boldsymbol{\delta}_k^{(j-1)})\}^{-1} \tag{10}$$

Here, j is the MCMC iteration number. The vector $\boldsymbol{a}$ represents the prior mean for the start of the random walk, i.e., $a_1 = \beta$ and zero otherwise. The matrix $\boldsymbol{\Lambda}$ is the covariance for the start of the random walk, i.e., $\Lambda_{11} = \sigma^2$ and zero otherwise.

The acceptance probability q for the $\boldsymbol{\delta}_k$ MH steps is given by:

$$q(\boldsymbol{\delta}_k^{(j-1)}, \boldsymbol{\delta}_k^{(j)}) = \min\left\{1, \frac{l_{sk}(\mathbf{y};\ \boldsymbol{\delta}_k^{(j)}, \epsilon_k) \cdot \mathrm{p}(\delta_k^{(j)} \mid \beta, \sigma, \rho) \cdot \mathrm{Normal}(\boldsymbol{\delta}_k^{(j)};\ \boldsymbol{m}_k^{(j-1)}, \mathrm{scale}_\delta \cdot \boldsymbol{C}_k^{(j-1)})}{l_{sk}(\mathbf{y};\ \boldsymbol{\delta}_k^{(j-1)}, \epsilon_k) \cdot \mathrm{p}(\delta_k^{(j-1)} \mid \beta, \sigma, \rho) \cdot \mathrm{Normal}(\boldsymbol{\delta}_k^{(j-1)};\ \boldsymbol{m}_k^{(j)}, \mathrm{scale}_\delta \cdot \boldsymbol{C}_k^{(j)})}\right\}$$

Here, scale_δ is a scaling factor that we set to $\mathrm{scale}_\delta = \mathrm{tune}_\delta \cdot 5.76/T$. The parameter tune_δ is then used to tune the acceptance rate to ≈ 0.23, the conventionally used acceptance rate for multidimensional MH (see [12] for details).

The function $l_{sk}(\mathbf{y};\ \boldsymbol{\delta}_k, \epsilon_k)$ is the data likelihood restricted to data in condition s assigned to prototype signature k:

$$l_{sk}(\mathbf{y};\ \boldsymbol{\delta}_k, \epsilon_k) = \prod_{g,i\,:\,z_{gsi}=k} \prod_{t,r} \text{Neg-Bin}(\mathsf{y}_{gsitr};\ \mu_k(t), \epsilon_k)$$

The conditonal probability $\mathrm{p}(\boldsymbol{\delta}_k \mid \beta, \sigma, \rho)$ is the product of the Normal priors on $\boldsymbol{\delta}_k$:

$$\mathrm{p}(\boldsymbol{\delta}_k \mid \beta, \sigma, \rho) = \mathrm{Normal}(\delta_{k1};\ \beta, \sigma) \prod_{\tau=2}^{T} \mathrm{Normal}(\delta_{k\tau};\ 0, \rho\sqrt{\Delta\tau})$$

The MH procedure for sampling $\boldsymbol{\delta}_k$ is then:

1. start with $\boldsymbol{\delta}_k$ sampled from the prior, i.e., $\delta_{k1} \sim \text{Normal}(\beta, \sigma)$ and $\delta_{kt} \sim \text{Normal}(0, \rho\sqrt{\Delta\tau})$

2. at iteration j, calculate the proposal distribution moments $\boldsymbol{m}_k^{(j)}$ and $\boldsymbol{C}_k^{(j)}$.

3. sample $\boldsymbol{\delta}_k^{(j)}$ from the proposal distribution $\text{Normal}(\boldsymbol{m}_k^{(j)}, \text{scale}_\delta \cdot \boldsymbol{C}_k^{(j)})$ and accept with probability $q(\boldsymbol{\delta}_k^{(j-1)}, \boldsymbol{\delta}_k^{(j)})$.

The MH procedure for the culture data model requires a slight modification of Equation 7. We must incorporate the offset term in the data transformation:

$$\widetilde{\mathsf{y}}_{gsitr}(\boldsymbol{\delta}_k) = \eta_k(t) + (\mathsf{y}_{gsitr} - \mu_k(t))h'(\mu_k(t)) - \ln \frac{o_{gsitr}}{\bar{o}} \tag{11}$$

6.3 Step 3: sampling prototype signature variables controlling NBD variance

We also use MH steps for sampling ϵ_k. In this case, because we are sampling a single variable, we use a simple proposal distribution of $\text{Normal}(\epsilon_k^{(j-1)}, \text{tune}_\epsilon \cdot \sqrt{\omega_{\epsilon 1}})$. The parameter tune_ϵ is used to tune the acceptance rate to ≈ 0.44, the conventionally used acceptance rate for single dimensional MH (see [12] for details). .

The acceptance probability for the ϵ_k MH steps is given by:

$$q(\epsilon_k^{(j-1)}, \epsilon_k^{(j)}) = \min\left\{1, \frac{l_{sk}(\mathbf{y};\ \boldsymbol{\delta}_k, \epsilon_k^{(j)}) \cdot \text{p}(\epsilon_k^{(j)} \mid \boldsymbol{\omega}_\epsilon) \cdot \text{Normal}(\epsilon_k^{(j)};\ \epsilon_k^{(j-1)}, \text{tune}_\epsilon \cdot \sqrt{\omega_{\epsilon 1}})}{l_{sk}(\mathbf{y};\ \boldsymbol{\delta}_k, \epsilon_k^{(j-1)}) \cdot \text{p}(\epsilon_k^{(j-1)} \mid \boldsymbol{\omega}_\epsilon) \cdot \text{Normal}(\epsilon_k^{(j-1)};\ \epsilon_k^{(j)}, \text{tune}_\epsilon \cdot \sqrt{\omega_{\epsilon 1}})}\right\}$$

6.4 Step 4: sampling the mean and variance for the prior on initial signature values

Sampling β and σ^2 is straightforward because these parameters use conjugate priors.

The prior for β is $\text{Normal}(m_\beta, v_\beta)$. Thus, the posterior distribution for β is:

$$\text{p}(\beta \mid \mathbf{y}, m_\beta, v_\beta) \propto \text{Normal}\left(\frac{\sum_{k=1}^K \delta_{k1}/\sigma^2 + m_\beta/v_\beta}{K/\sigma^2 + 1/v_\beta}, \frac{1}{\sqrt{K/\sigma^2 + 1/v_\beta}}\right)$$

Here K is the number of non-empty components.

The prior for σ^2 is $\text{Inv-}\chi^2(v_\sigma, \xi_\sigma)$. Thus, the posterior distribution for σ^2 is:

$$\text{p}(\sigma^2 \mid \mathbf{y}, v_\sigma, \xi_\sigma) \propto \text{Inv-Gamma}\left(\frac{\xi_\sigma + K}{2}, \frac{\sum_{k=1}^K (\delta_{k1} - \beta)^2 + \xi_\sigma v_\sigma}{2}\right)$$

6.5 Step 5: sampling the random walk variance

Sampling ρ^2, the random walk variance, is straightforward because ρ^2 has a conjugate prior of $\text{Inv-}\chi^2(v_\rho, \xi_\rho)$. Thus, the posterior distribution for ρ^2 is:

$$\text{p}(\rho^2 \mid \mathbf{y}, v_\rho, \xi_\rho) \propto \text{Inv-Gamma}\left(\frac{\xi_\rho + TK}{2}, \frac{\sum_{t=2}^T \sum_{k=1}^K \delta_{kt}^2/\Delta t + \xi_\rho v_\rho}{2}\right)$$

6.6 Step 6: sampling the concentration parameter

For sampling the concentration parameter α, we use an efficient auxiliary variable sampling method developed by Escobar and West [13].

It can be shown that the conditional distribution for α is given by (see [14]):

$$\mathrm{p}(\alpha \mid K, N, \boldsymbol{\omega}_\alpha) \propto \alpha^{\omega_{\alpha 1}+K-1} e^{-\omega_{\alpha 2}\alpha} \mathrm{B}(\alpha, N)$$

Here K is the number of non-empty prototype signatures, N is the number of input data items, and $\mathrm{B}(\cdot,\cdot)$ is the standard Beta function defined as:

$$\mathrm{B}(u, v) = \frac{\Gamma(u)\Gamma(v)}{\Gamma(u+v)} = \int_0^1 \phi^{u-1}(1-\phi)^{v-1} d\phi$$

We can then write $\mathrm{p}(\alpha \mid K, N)$ as a marginalization over an auxiliary variable ϕ:

$$\mathrm{p}(\alpha \mid K, N, \boldsymbol{\omega}_\alpha) \propto \int_0^1 \mathrm{p}(\alpha, \phi \mid K, N, \boldsymbol{\omega}_\alpha) d\phi$$

$$\mathrm{p}(\alpha, \phi \mid K, N, \boldsymbol{\omega}_\alpha) \propto \alpha^{\omega_{\alpha 1}+K-1} e^{-\omega_{\alpha 2}\alpha} \phi^{\alpha-1}(1-\phi)^{N-1}$$

From the joint distribution, we can see that:

$$\mathrm{p}(\alpha \mid \phi, K, N, \boldsymbol{\omega}_\alpha) \propto \mathrm{Gamma}(\alpha;\ \omega_{\alpha 1} + K - 1, \omega_{\alpha 2} - \ln\phi) \tag{12}$$

$$\mathrm{p}(\phi \mid \alpha, K, N) \propto \mathrm{Beta}(\phi;\ \alpha, N) \tag{13}$$

Thus, by alternating sampling of α and ϕ according to Equations 12 and 13 above, we sample from the posterior for α in the MCMC sampling iterations.

7 Detecting changes in signatures from infected subjects versus uninfected controls

As discussed in Section 2, MC-TIMME is a fully Bayesian model, which allows us to directly compute posterior probabilities of interest. To detect whether the behavior of a taxon g in site s changes in infected subjects versus uninfected controls, we are interested in the probability p_{gs} of the taxon's time-series of counts in the two conditions being assigned to different prototype signatures. The samples from our MCMC inference method described in Section 6 allow us to readily estimate this probability:

$$\mathrm{p}_{gs} = \frac{\sum_{j=1}^{M} I(z_{gs1}^{(j)} \neq z_{gs2}^{(j)})}{N_{\text{MCMC}}}$$

Here, N_{MCMC} is the number of MCMC iterations and $z_{gsi}^{(j)}$ is the assignment of taxon g in site s and condition i (infected or uninfected) at MCMC iteration j.

We also characterize the posterior distribution of assigned prototype signature shape parameters with median and 95% credible interval statistics. The estimated centiles for the posterior distribution of assigned prototype signature shape parameters for taxon g in site s in condition i are calculated as:

$$\underset{j=1,\ldots,N_{\text{MCMC}}}{\text{centile}(c)} \left\{ exp(\sum_{\tau=0}^{t} \delta^{(j)}_{z_{gsi}t}) \right\} \tag{14}$$

Our criteria for detecting differences in infected and control signatures for taxon g in site s was to require $1 - p_{gs} < 0.05$ and that the 95% credible intervals for the infected and uninfected signatures not overlap for at least two time-points. The rationale for requiring changes for at least two time-points was that in the present study we were interested in taxa with sustained alternations in behavior, rather than those just exhibiting a single large change. Note that for detecting differences in behavior of taxa, MC-TIMME takes into account the entire time-series as well as behavior of other taxa. This provides significant advantages over a simple strategy of performing many independent statistical tests.

To estimate the false detection rate, we performed permutation testing. Each trial consisted of randomly permuting data for each taxon g across time-points and replicates, and then running MC-TIMME and detecting changes between infected and uninfected signatures as described above. The permuted data approximates a "null distribution" in which no taxon exhibits significant changes in the infected versus the uninfected state. We performed 100 trials, and detected an average of 4% of taxa as significantly changing, suggesting a false positive rate of $< 5\%$ on our dataset.

8 Systems measures

8.1 Site, condition and time dependent entropy

We use the estimated prototype signatures and taxa assignments to signatures to calculate the entropy of the distribution formed by the fraction of counts assigned to each taxa at each time point. The fraction of counts assigned to taxon g in site s in condition i at time t and for MCMC sample j is given by:

$$f^{(j)}_{gsit} = \frac{exp(\sum_{\tau=0}^{t} \delta^{(j)}_{z_{gsi}t})}{\sum_{g'} exp(\sum_{\tau=0}^{t} \delta^{(j)}_{z_{g'si}t})}$$

Here, $z^{(j)}_{gsi}$ is the assignment of taxon g in site s and condition i (infected or uninfected) at MCMC iteration j to a particular prototype signature, and $\delta^{(j)}_{z_{gsi}t})$ is the shape parameter at time t for that signature.

The entropy for site s in condition i at time t for MCMC sample j is thus:

$$H^{(j)}(s,i,t) = -\sum_{g} f^{(j)}_{gsit} \ln f^{(j)}_{gsit}$$

The median and credible intervals for entropy distributions are then calculated using the estimated posterior distribution from the MCMC samples.

8.2 Time to recovery at each site

We define the time to recovery at a specific anatomic site as the latest time at which a weighted measure of changing OTUs is less than 5%. We define the weighted measure as follows. The median fraction of counts assigned to taxon g in site s in condition i at time t is given by:

$$\overline{f}_{gsit} = \frac{\overline{\mu}_{gsi}(t)}{\sum_{g'} \overline{\mu}_{g'si}(t)}$$

Here, $\overline{\mu}_{gsi}(t)$ is the median of the assigned signature for taxa g in site s in condition i at time t as defined in equation 14.

Let I_{gst} denote an indicator variable that is equal to one (and zero otherwise) if the 95% credible intervals as defined in Section 7 for taxon g in site s at time t do not overlap for the infected and uninfected conditions, i.e., the indicator is equal to one if there is a change between the two conditions at that time point.

The weighted measure of changing OTUs in site s at time t is then defined as:

$$\sum_g I_{gst} \sqrt{\overline{f}_{gs1t} \overline{f}_{gs2t}}$$

This measure may be viewed as the truncated Hellinger distance [15] between the distributions of estimated taxa counts in the infected and uninfected conditions. The Hellinger distance is a general measure of distance between two probability distributions, with a particularly simple form in the case of multinomial distributions. We use a truncated version that only sums up the contributions of taxa that are detected as changing between the two conditions. Thus, the measure will be zero if no taxa are detected as changing, and can attain a maximum of one if all taxa are detected as changing and the difference between their proportions in the infected and uninfected states is maximal.

9 Time-maps

The purpose of time-maps is to visualize cascading changes in taxa behavior with infection (relative to the uninfected baseline) in a particular site, as well as to visualize groups of functionally related taxa in that site. Each row in the time-map visualizes a particular taxon g in site s across time.

We define the change for taxa g in site s at time t as:

$$\Psi(g, s, t) = \mu_{z_{gs2}}(t) - \mu_{z_{gs1}}(t) \tag{15}$$

Here, z_{gs1} is the index of the prototype signature that taxa g in site s is assigned to in the uninfected state, and z_{gs2} is the corresponding index for the infected state.

The visualized function is a normalized version of $\Psi(g, s, t)$, in which we normalize by the standard deviation of the vector $(\Psi(g, s, 1), \ldots, \Psi(g, s, T))$. The visualized color at a time-point t is red if $\Psi(g, s, t) > 0$ and blue if $\Psi(g, s, t) < 0$. The intensity of the color at each time-point t is scaled based on the magnitude of $\Psi(g, s, t)$, and standardized across the entire figure by the upper and lower 95% percentile values of $\Psi(\cdot, s, \cdot)$ over all time-points and taxa. To improve the visualization, we linearly interporate between time-points. Further, for time-points (or interpolated regions) in which the 95% credible intervals

of infected and uninfected assigned prototype signatures overlap, we quench the signal by 80% to indicate regions in which the significance of the change is uncertain.

Each taxon g is then assigned a vector consisting of: 1) the average time of maximal change for its functional group, 2) the index of the functional group to which the taxa belongs, 3) the time of maximal change for the taxa. The rows in the figure are then sorted by this vector. The net effect is that the rows are organized into functional groups, with the groups exhibiting average maximal changes earliest at the top of the figure, and taxa within groups sorted by their times of maximal change.

References

[1] Gerber, G. K., Onderdonk, A. B. & Bry, L. Inferring Dynamic Signatures of Microbes in Complex Host Ecosystems. *PLoS Computational Biology* **8**, e1002624 (2012).

[2] Anders, S. & Huber, W. Differential expression analysis for sequence count data. *Genome Biology* **11**, R106 (2010).

[3] Robinson, M. D., McCarthy, D. J. & Smyth, G. K. edgeR: a Bioconductor package for differential expression analysis of digital gene expression data. *Bioinformatics (Oxford, England)* **26**, 139–40 (2010).

[4] Houseman, E. A., Coull, B. & Shine, J. P. A nonstationary negative binomial time series with time-dependent covariates: Enterococcus counts in Boston Harbor (2005).

[5] Stephens, M. & Balding, D. J. Bayesian statistical methods for genetic association studies. *Nature Reviews Genetics* **10**, 681–90 (2009).

[6] Scott, J. G. & Berger, J. O. An exploration of aspects of Bayesian multiple testing. *Journal of Statistical Planning and Inference* 2144–2162 (2006).

[7] Sethuraman, J. A Constructive Definition of Dirichlet Priors. *Statistica Sinica* **4**, 639–650 (1994).

[8] Antoniak, C. Mixtures of Dirichlet processes with applications to Bayesian nonparametric problems. *Annals of Statistics* **2**, 1152–1174 (1974).

[9] Rasmussen, C. The Infinite Gaussian Mixture Model. In *Neural Information Processing Systems (NIPS)* (2000).

[10] Clayton, D. Generalized linear mixed models. In Gilks, W., Richardson, S. & Spiegelhalter, D. (eds.) *Markov Chain Monte Carlo in Practice*, 275–301 (Chapman & Hall/CRC, 1996).

[11] Gamerman, D. Sampling from the posterior distribution in generalized linear mixed models. *Statistics and Computing* **7**, 57–68 (1997).

[12] Gelman, A., Carlin, J., Stern, H. & Rubin, D. *Bayesian Data Analysis* (Chapman & Hall/CRC, 2004).

[13] Escobar, M. D. & West, M. Bayesian Density Estimation and Inference Using Mixtures. *Journal of the American Statistical Association* **90**, 577–588 (1995).

[14] Navarro, D. J., Griffiths, T. L., Steyvers, M. & Lee, M. D. Modeling individual differences using Dirichlet processes. *Journal of Mathematical Psychology* **50**, 101–122 (2006).

[15] Basu, A., Harris, I. R. & Basu, S. Minimum distance estimation: The approach using density-based distances. In Maddala, G. S. & Rao, C. R. (eds.) *Handbook of Statistics*, vol. 15, 21–48 (North-Holland, 1997).
